# Dietary patterns, lung function and asthma in childhood: a longitudinal study

**DOI:** 10.1186/s12931-023-02383-9

**Published:** 2023-03-16

**Authors:** Mohammad Talaei, Pauline M. Emmett, Raquel Granell, Hossein Tabatabaeian, Kate Northstone, Anna Bergström, Seif O. Shaheen

**Affiliations:** 1 Wolfson Institute of Population Health, Barts and The London School of Medicine and Dentistry, Queen Mary University of London, Charterhouse Square, London, EC1M 6BQ UK; 2 Centre for Academic Child Health, Population Health Sciences, Bristol Medical School, University of Bristol, Bristol, UK; 3 MRC Integrative Epidemiology Unit (IEU), Population Health Sciences, Bristol Medical School, University of Bristol, Bristol, UK; 4 Cancer Science Institute of Singapore, National University of Singapore, Singapore, Singapore; 5 Population Health Sciences, Bristol Medical School, University of Bristol, Bristol, UK; 6 Institute of Environmental Medicine, Karolinska Institute, Stockholm, Sweden; 7 Centre for Occupational and Environmental Medicine, Region Stockholm, Stockholm, Sweden

**Keywords:** Dietary pattern, Lung function, Asthma, Childhood, Diet, ALSPAC

## Abstract

**Background:**

Longitudinal epidemiological data are scarce examining the relationship between dietary patterns and respiratory outcomes in childhood. We investigated whether three distinct dietary patterns in mid-childhood were associated with lung function and incident asthma in adolescence.

**Methods:**

In the Avon Longitudinal Study of Parents and Children, ‘processed’, ‘traditional’, and ‘health-conscious’ dietary patterns were identified using principal components analysis from food frequency questionnaires at 7 years of age. Post-bronchodilator forced expiratory volume in 1 s (FEV_1_), forced vital capacity (FVC), and forced expiratory flow at 25–75% of FVC (FEF_25–75_) were measured at 15.5 years and were transformed to z-scores based on the Global Lung Function Initiative curves. Incident asthma was defined by new cases of doctor-diagnosed asthma at age 11 or 14 years.

**Results:**

In multivariable-adjusted models, the ‘health-conscious’ pattern was positively associated with FEV_1_ (regression coefficient comparing top versus bottom quartile of pattern score 0.16, 95% CI 0.01 to 0.31, P for trend 0.04) and FVC (0.18, 95% CI 0.04 to 0.33, P for trend 0.02), while the ‘processed’ pattern was negatively associated with FVC (− 0.17, 95% CI − 0.33 to − 0.01, P for trend 0.03). Associations between the ‘health-conscious’ and ‘processed’ patterns and lung function were modified by *SCGB1A1* and *GPX4* gene polymorphisms. We found no evidence of an association between the ‘traditional’ pattern and lung function, nor between any pattern and FEF_25–75_ or incident asthma.

**Conclusions:**

A ‘health-conscious’ diet in mid-childhood was associated with higher subsequent lung function, while a diet high in processed food was associated with lower lung function.

**Supplementary Information:**

The online version contains supplementary material available at 10.1186/s12931-023-02383-9.

## Key messages

**What** **is** **already** **known** **on** **this** **topic:** Evidence on the potential role of dietary patterns in the aetiology of childhood asthma and impaired lung function is very limited, and longitudinal studies are scarce.

**What** **this** **study** **adds:** We found that children with a higher score on the ‘health-conscious’ pattern had higher lung function in adolescence, whereas children with a higher score on the ‘processed’ pattern had lower lung function. These relationships were not explained by potential mediators such as adiposity, dietary fibre, vitamin D, long-chain omega-3, and antioxidants, but there was evidence that they may partly depend on genetic make-up. We did not find any evidence of association with incident asthma.

**How this study might affect research, practice or policy:** Beyond possible effects of individual foods and nutrients, dietary patterns may have an additional influence on lung function growth. If our findings are replicated, this might inform potential dietary interventional studies to improve lung health.

## Introduction

Accumulating evidence has shown the importance of maximal lung function attainment as lung function in early adulthood is linked to subsequent comorbidities and early mortality [1, 2]. Childhood may serve as a window of susceptibility to exposures with the potential to influence lifelong respiratory health, and diet may have a role [3]. Whilst the traditional single food/nutrient approach is useful, dietary pattern analysis examines the overall diet, paralleling more closely the real world, and takes into account the complex interactions within foods and the correlation between nutrients [4]. There are two major approaches to dietary patterns: a priori which is predefined and based on prior knowledge, and a posteriori (empirical) which describes eating habit variation in the population without any prior hypothesis [4].

Previous studies on the link between various dietary patterns and asthma or lung function in childhood were mostly cross-sectional or case–control, with few exceptions that were either in children with asthma (not the general population) [5], in very early childhood [6, 7], or with very short follow-up [7]. The lack of prospective studies is a major limitation and there is a pressing need for longitudinal studies in children [8, 9]. Most previous studies used an a priori approach, focusing on a Mediterranean diet, and suggested that higher adherence to such a diet was associated with a lower risk of prevalent asthma [10–13], though not always [14], or with better lung function [5, 12]. However, culture-driven patterns like the Mediterranean diet need to be adapted for other populations to consider the many potentially confounding differences between populations [15]. Identifying dietary patterns a posteriori might be the best way to reveal the healthiest type of diet in a population [15] as, unlike the a priori approach, it is not limited by our current knowledge for decisions on the selection of components and on arbitrary cut-offs to define adherence [4, 10]. To our knowledge, this approach was only used in two studies; in a cross-sectional study that reported a positive association between a ‘Western’ pattern and asthma in school-age children [16] and in a birth cohort study which reported that an empirically derived ‘Western’ pattern at 14 months was not associated with wheeze at 3–4 years of age [6].

In this study, we have explored the relations of three distinct empirical dietary patterns at 7 years of age, previously derived using principal component analysis (PCA; a common method for deriving dietary patterns a posteriori) [17], with lung function in adolescence and incident asthma. To inform mechanisms, we have explored potential mediators, and whether associations were modified by polymorphisms linked to antioxidant defence or to club cell secretory protein, which is associated with lung function growth in childhood [18]; biologically plausible interactions with gene variants could strengthen causal inference.

## Methods

### Study population

We used the Avon Longitudinal Study of Parents and Children (ALSPAC), a population-based birth cohort that recruited pregnant women resident in Avon, UK (15,454 pregnancies) with due dates between April 1991 and December 1992. Participants have been followed up since birth with questionnaires and objective measures in research clinics. The study protocol has been described previously [19, 20] and further information can be found at www.alspac.bris.ac.uk, which contains details of all available data (http://www.bristol.ac.uk/alspac/researchers/our-data/).

### Exposure assessment

We used three previously defined dietary patterns, ‘health-conscious’, ‘traditional’, and ‘processed’, which were derived using PCA on 57 standardised food items [17] collected by food frequency questionnaire (FFQ) at ~ 7 years (81 months) of age [21]. For each child, a score was calculated for each pattern (uncorrelated with other patterns because of orthogonal rotation); a higher score suggesting that the dietary pattern describes the type of diet consumed by that child more closely (summary of factor loadings in Additional file 1: Table S1 [17]). These three components explained 3.8%, 7.0%, and 7.3% of the variance in the data, respectively [22].

The daily intake of each food group was estimated using standard portion sizes based on typical consumption patterns in Britain [23] adapted for the age of children. Approximate daily intakes for total energy and nutrients were calculated by multiplying estimated food intake (g/day) by their estimated nutrient content from UK food composition tables [24] and summing this across all the foods consumed. We also estimated the total antioxidant capacity (TAC) of the diet using the average oxygen radical absorbance capacity content of foods [25]. Nutrient intakes and TAC were adjusted for total energy intake using the residual method [26].

### Outcome assessment

Lung function was assessed by spirometry (Vitalograph 2120; Vitalograph¸ Maids Moreton, UK) at 15.5 years according to American Thoracic Society standards [27], after withholding short-acting bronchodilators for at least 6 h, and long-acting bronchodilators and theophyllines for at least 24 h. The best of three reproducible flow–volume curves was used to measure forced expiratory volume in 1 s (FEV_1_), forced vital capacity (FVC), and forced expiratory flow at 25–75% of FVC (FEF_25–75_), before and 15 min after administration of 400 mg of salbutamol. We transformed these lung function measurements to z-scores based on the Global Lung Function Initiative (GLI) curves, adjusting for age, height, and ethnicity, and separately by sex [28], using the GLI R macro [available from https://github.com/thlytras/rspiro] [29]. ‘Post-bronchodilator’ FEV_1_ and FEF_25-75_ are more likely to reflect growth and calibre of the airways rather than their tone.

Incident asthma was our second primary outcome of interest. We defined current doctor-diagnosed asthma at ~ 7.5 years (91 months), ~ 11 years (128 months), and ~ 14 years (166 months) if mothers responded positively to the question “Has a doctor ever actually said that your study child has asthma?” and to at least one of the questions which asked if the child had had wheezing, wheezing and whistling in the chest, asthma, or asthma medication in the last 12 months. There was a good agreement between parental reports of a doctor’s diagnosis of asthma in ALSPAC and GP-recorded diagnosis (sensitivity 88.5%, specificity 95.7%) [30]. We defined incident asthma as new cases of current doctor-diagnosed asthma at age 11 or 14 years.

### Antioxidant gene polymorphism selection

As the ‘health-conscious’ and ‘traditional’ dietary patterns are likely to be antioxidant-rich (and the ‘processed’ pattern antioxidant-poor) [31], we hypothesised that associations with outcomes may be modified by common antioxidant gene variants. These include: glutathione-S-transferase (GST) variants, namely null deletions in *GSTT1* and *GSTM1*, and a missense substitution in *GSTP1* (G313A, Ile105Val, rs1695) [32, 33]; also polymorphisms in the glutathione peroxidase 4 (*GPX4*) gene (rs713041) [34], the aryl hydrocarbon receptor (*AHR*) gene (rs2066853) [35] which is activated by compounds in cruciferous vegetables [36], and the secretoglobin family 1A member 1 (*SCGB1A1*) gene (rs3741240), which encodes the club cell secretory protein (CC16). The latter single nucleotide polymorphism (SNP) was shown to have the strongest correlation with serum levels of CC16 protein in a genome-wide association study [37]. CC16 is an airway epithelial biomarker, which has anti-inflammatory and antioxidant properties [38] and is positively associated with lung function growth in childhood [18] (summary of genotyping in Additional file 1: Table S2).

### Statistical analysis

Among 8,135 children with data on dietary patterns at 7 years (excluding children with more than 10 missing items or implausible total energy intake: < 15,000 kJ/w or > 140,000 kJ/w), data on post-bronchodilator lung function measures at 15.5 years and incident asthma were complete for 2,950 − 3,085 (depending on the specific measure) and 4,491 participants, respectively (see Additional file 1: Fig. S1). We examined the associations of dietary pattern scores (in quartiles and per standard deviation) with lung function measures using linear regression analysis and with incident asthma using logistic regression analysis. We tested for linear trends across quartiles by assigning median values to each of the four categories and then using it as a continuous ordinal variable in the models. The shape of relationships was assessed using restricted cubic spline analysis. We selected known potential confounding factors from the existing literature [39] and by using a directed acyclic graph approach [40]; these included multiple individual and area-based indicators of socioeconomic status (SES) (see Additional file 1: Fig. S2).

By further adjustment in separate models, we explored if the associations could be explained by potential mediators including dietary antioxidants, vitamins, minerals, long-chain omega-3 fatty acids from fish, and fibre (all as quartiles), as well as body mass index (BMI) and fat mass index (FMI) at 9 years of age.

We carried out stratified analyses, a priori, to explore the potential modification of dietary associations by sex, prevalent doctor-diagnosed asthma at 7.5 years, exposure to oxidative stress including maternal smoking when the child was 7 years of age (none, 1–14/day, and ≥ 15/day) and outdoor particulate matter up to 10 µm in size (PM_10_, tertiles) at age 7 years, and by antioxidant gene variants listed above (Additional file 1: Table S1). Potential interactions by these factors were examined by testing their cross-product terms with quartiles of each dietary pattern in regression models.

We also carried out several sensitivity analyses, a priori, including further adjustment for other potential confounders, inverse probability weighting to correct for potential loss to follow-up bias [41], and a multilevel modelling approach considering measurements at 8 and 15.5 years of age (pre-bronchodilator as they were the only available measures at 8 years) using panel data analysis (xtreg, random effect). All statistical analyses were carried out using Stata version 14.2 (StataCorp, College Station, TX, USA). Further details about dietary patterns, TAC, genotyping, covariates, multivariable models, sensitivity analyses, and restricted cubic spline analysis are explained in the online supplementary materials (Additional file 1).

## Results

Table 1 shows that children with a higher score on the ‘health-conscious’ dietary pattern were more likely to live in rural and least deprived areas, more likely to have mothers with a higher level of education, to have been exclusively breastfed, to have a history of food allergy and higher hours of vigorous physical exercise, but less likely to have a smoking mother. They also had higher dietary TAC and higher intake of vitamins A, C, D, and E, and β-carotene, zinc, selenium, fibre, and long-chain omega-3 from fish, and were more likely to consume dietary supplements. All these relationships were in the opposite direction for the ‘processed’ pattern. The ‘health-conscious’ pattern was associated with lower FMI at 9 years, while the ‘processed’ pattern was associated with higher BMI at 9 years. The ‘traditional’ dietary pattern showed fewer relationships with child or mother characteristics but showed similar associations with dietary nutrients as the ‘health-conscious’ pattern (see Additional file 1: Tables S3, S4, and S5 for further detail).

**Table 1:** Participant characteristics according to top and bottom quartiles of dietary pattern score at 7 years of age

	‘Processed’		‘Traditional’		‘Health-conscious’	
	Q1	Q4	Q1	Q4	Q1	Q4
n (%)	1449 (26.8)	1222 (22.6)	1378 (25.5)	1343 (24.9)	1267 (23.5)	1385 (25.6)
Male, n (%)	678 (46.8)	634 (51.9)	737 (53.5)	619 (46.1)	666 (52.6)	683 (49.3)
Index of Multiple Deprivation, n (%)						
Q1: Least deprived	520 (40.5)	290 (26.0)	399 (32.2)	394 (32.5)	294 (25.5)	471 (38.6)
Q5: Most deprived	144 (11.2)	169 (15.1)	189 (15.2)	154 (12.7)	169 (14.6)	154 (12.6)
Place of residence, n (%)						
Urban	1006 (69.4)	979 (80.1)	1070 (77.6)	1008 (75.1)	1009 (79.6)	984 (71.0)
Rural	295 (20.4)	144 (11.8)	181 (13.1)	214 (15.9)	153 (12.1)	247 (17.8)
Older siblings, n (%)	718 (49.6)	646 (52.9)	704 (51.1)	713 (53.1)	640 (50.5)	717 (51.8)
Younger siblings, n (%)	749 (51.7)	659 (53.9)	695 (50.4)	716 (53.3)	663 (52.3)	717 (51.8)
Breastfeeding by the 3rd month, n (%)						
Never	131 (9.0)	260 (21.3)	230 (16.7)	183 (13.6)	288 (22.7)	93 (6.7)
Stopped/Non-exclusive	630 (43.5)	600 (49.1)	622 (45.1)	640 (47.7)	617 (48.7)	594 (42.9)
Exclusive	620 (42.8)	299 (24.5)	456 (33.1)	458 (34.1)	310 (24.5)	617 (44.5)
Vigorous physical activity, n (%)						
< 3 times a week	590 (40.7)	526 (43.0)	634 (46.0)	524 (39.0)	585 (46.2)	510 (36.8)
4–6 times a week	361 (24.9)	243 (19.9)	276 (20.0)	316 (23.5)	235 (18.5)	349 (25.2)
Daily	146 (10.1)	118 (9.7)	130 (9.4)	142 (10.6)	106 (8.4)	168 (12.1)
BMI, kg/m^2^	16.1 ± 1.9	16.3 ± 2.1	16.1 ± 2.0	16.2 ± 1.9	16.1 ± 2.0	16.1 ± 1.9
BMI at 9 years, kg/m^2^	17.4 ± 2.6	17.7 ± 2.9	17.6 ± 2.9	17.7 ± 2.7	17.6 ± 2.9	17.4 ± 2.6
FMI at 9 years, kg/m^x^	1.23 ± 0.7	1.28 ± 0.8	1.25 ± 0.8	1.30 ± 0.8	1.27 ± 0.8	1.20 ± 0.7
History of food allergy, n (%)	324 (22.4)	183 (15.0)	233 (16.9)	230 (17.1)	182 (14.4)	289 (20.9)
Atopy, n (%)	224 (20.6)	180 (20.6)	209 (20.5)	189 (18.7)	175 (19.6)	231 (21.3)
Season of dietary information collection^†^, n (%)						
Winter	411 (28.4)	285 (23.3)	333 (24.2)	353 (26.3)	338 (26.7)	331 (23.9)
Spring	424 (29.3)	347 (28.4)	419 (30.4)	383 (28.5)	397 (31.3)	411 (29.7)
Summer	354 (24.4)	412 (33.7)	407 (29.5)	368 (27.4)	340 (26.8)	410 (29.6)
Autumn	243 (16.8)	166 (13.6)	202 (14.7)	226 (16.8)	187 (14.8)	210 (15.2)
Any dietary supplement use, n (%)	539 (37.2)	371 (30.4)	521 (37.8)	406 (30.2)	302 (23.8)	565 (40.8)
Total energy intake, kJ/day	6592 ± 1417	9046 ± 1773	6980 ± 1642	8457 ± 1838	6931 ± 1540	8319 ± 1899
Total antioxidant capacity, μmol of TE/d	7894 ± 1937	7297 ± 1997	7173 ± 1982	8003 ± 2002	6826 ± 1612	8359 ± 2116
Vitamin C intake, mg/d	83.8 ± 31.1	70.7 ± 32.5	69.2 ± 30.3	88.3 ± 32.8	64.1 ± 24.5	90.3 ± 34.4
Vitamin D intake, mg/d	2.93 ± 0.8	2.71 ± 0.8	2.70 ± 0.8	3.03 ± 0.9	2.78 ± 0.7	2.92 ± 0.9
Vitamin E intake, mg/d	9.91 ± 2.9	9.65 ± 3.0	9.98 ± 3.2	9.71 ± 2.9	9.62 ± 2.9	9.98 ± 3.0
β-carotene intake, μg/d	2326 ± 852	1635 ± 811	1516 ± 728	2508 ± 918	1790 ± 753	2199 ± 947
Zinc intake, mg/d	6.81 ± 0.9	6.00 ± 1.0	5.83 ± 0.9	7.00 ± 1.0	6.19 ± 0.9	6.68 ± 1.0
Selenium intake, μg/d	68.7 ± 14.2	59.8 ± 14.0	62.1 ± 14.0	68.2 ± 15.1	62.6 ± 12.1	68.6 ± 15.9
VLC n-3 PUFA intake from fish, mg/d	98.0 ± 96.9	65.5 ± 81.2	57.0 ± 61.9	113 ± 115	57.9 ± 55.4	111 ± 115
Preformed vitamin A intake, μg/d	485 ± 125	429 ± 182	448 ± 135	469 ± 158	442 ± 127	469 ± 158
Dietary fibre intake, g/d	12.9 ± 2.5	10.3 ± 2.2	10.7 ± 2.6	12.4 ± 2.5	10.2 ± 1.7	13.2 ± 2.7
Maternal smoking, n (%)						
No	1203 (83.0)	918 (75.1)	1098 (79.7)	1049 (78.1)	974 (76.9)	1130 (81.6)
Yes	193 (13.3)	248 (20.3)	235 (17.1)	231 (17.2)	244 (19.3)	201 (14.5)
**Parental factors in pregnancy**						
Maternal age, year	30.2 ± 4.4	28.3 ± 4.4	29.6 ± 4.5	29.0 ± 4.5	28.6 ± 4.5	30.2 ± 4.5
Maternal education, n (%)						
Secondary or vocational	199 (13.7)	322 (26.4)	288 (20.9)	253 (18.8)	379 (29.9)	147 (10.6)
O level	374 (25.8)	498 (40.8)	459 (33.3)	478 (35.6)	522 (41.2)	354 (25.6)
A level or degree	864 (59.6)	381 (31.2)	613 (44.5)	593 (44.2)	346 (27.3)	869 (62.7)
Housing tenure, n (%)						
Mortgaged/owned	1220 (84.2)	987 (80.8)	1115 (80.9)	1133 (84.4)	1042 (82.2)	1154 (83.3)
Council rented	65 (4.5)	113 (9.2)	93 (6.7)	79 (5.9)	118 (9.3)	59 (4.3)
Non-council rented	103 (7.1)	65 (5.3)	114 (8.3)	67 (5.0)	55 (4.3)	122 (8.8)
Financial difficulty, n (%)						
No	1226 (84.6)	1000 (81.8)	1154 (83.7)	1110 (82.7)	1087 (85.8)	1147 (82.8)
Yes	216 (14.9)	216 (17.7)	215 (15.6)	229 (17.1)	175 (13.8)	229 (16.5)
Maternal ethnicity, n (%)						
White	1399 (96.5)	1189 (97.3)	1337 (97.0)	1299 (96.7)	1235 (97.5)	1335 (96.4)
Non-white	33 (2.3)	13 (1.1)	26 (1.9)	23 (1.7)	10 (0.8)	33 (2.4)
Maternal history of atopy, n (%)						
No	713 (49.2)	647 (52.9)	716 (52.0)	710 (52.9)	689 (54.4)	684 (49.4)
Yes	688 (47.5)	535 (43.8)	619 (44.9)	580 (43.2)	537 (42.4)	644 (46.5)
Paternal history of atopy, n (%)						
No	644 (44.4)	540 (44.2)	565 (41.0)	578 (43.0)	552 (43.6)	609 (44.0)
Yes	468 (32.3)	344 (28.2)	452 (32.8)	394 (29.3)	373 (29.4)	440 (31.8)
Maternal ‘Processed’ dietary pattern score	− 0.42 ± 0.7	0.21 ± 1.0	− 0.15 ± 1.0	− 0.08 ± 0.9	− 0.00 ± 1.0	− 0.26 ± 0.9
Maternal ‘Traditional’ dietary pattern score	0.05 ± 1.0	− 0.03 ± 1.0	− 0.24 ± 0.9	0.35 ± 1.1	− 0.12 ± 0.9	0.17 ± 1.1
Maternal ‘Healthy’ dietary pattern score	0.46 ± 0.9	0.01 ± 0.9	0.15 ± 1.0	0.31 ± 1.0	− 0.27 ± 0.8	0.73 ± 0.9

Children included in incident asthma or lung function analysis (n = 5400). The characteristics in the table are at around 7 years of child age unless otherwise stated or listed under the ‘Parental factors in pregnancy’. Numbers are mean ± SD unless otherwise specified

BMI: Body mass index; FMI: Fat mass index; TE: Trolox equivalents; VLC n− 3 PUFA: very− long-chain ω-3 polyunsaturated fatty acids

Further details including values in middle quartiles of dietary patterns, P values, and missing categories in some items are presented in Additional file 1: Tables S3–S5

### Lung function

After adjusting for potential confounders, including several indicators of SES, we found a linear positive association between the ‘health-conscious’ pattern score and FEV_1_ and FVC (P for non-linearity 0.11 and 0.14, respectively) after a mean (± SD) 8.7 (± 0.3) years of follow-up, but no evidence of association with FEV_1_/FVC or FEF_25-75_ (Table 2). Conversely, there was a non-linear negative association between the ‘processed’ pattern and FVC (P for non-linearity 0.01), and weak evidence for a negative association with FEV_1_ (P for non-linearity 0.03), but no evidence of association with FEV_1_/FVC or FEF_25-75_. The ‘traditional’ dietary pattern was not associated with any lung function measure.

**Table 2:** Linear regression coefficients (95% confidence interval) for post-bronchodilator lung function measures (z scores) at 15.5 years according to quartiles of dietary pattern scores at 7 years, adjusted for potential confounders

	Quartiles of dietary pattern score				P for	Per SD
	Q1	Q2	Q3	Q4	trend^a^	
‘Health-conscious’ dietary pattern						
FEV_1_						
Model 1	0.00	0.08 (− 0.06, 0.22)	0.09 (− 0.05, 0.23)	0.17 (0.03, 0.31)	0.02	0.05 (0.00, 0.10)
Model 2	0.00	0.08 (− 0.06, 0.22)	0.09 (− 0.05, 0.23)	0.16 (0.01, 0.31)	0.04	0.05 (− 0.01, 0.10)
FVC						
Model 1	0.00	0.08 (− 0.05, 0.22)	0.08 (− 0.06, 0.21)	0.20 (0.06, 0.33)	0.006	0.07 (0.02, 0.12)
Model 2	0.00	0.08 (− 0.06, 0.21)	0.08 (− 0.06, 0.21)	0.18 (0.04, 0.33)	0.02	0.06 (0.01, 0.11)
FEV_1_/FVC ratio						
Model 1	0.00	− 0.02 (− 0.14, 0.10)	− 0.00 (− 0.12, 0.12)	− 0.05 (− 0.17, 0.07)	0.41	− 0.03 (− 0.08, 0.01)
Model 2	0.00	− 0.02 (− 0.14, 0.10)	0.01 (− 0.12, 0.13)	− 0.06 (− 0.19, 0.07)	0.41	− 0.04 (− 0.09, 0.01)
FEF_25–75_						
Model 1	0.00	0.08 (− 0.04, 0.20)	0.06 (− 0.06, 0.18)	0.12 (− 0.01, 0.24)	0.10	0.04 (− 0.01, 0.08)
Model 2	0.00	0.08 (− 0.04, 0.20)	0.05 (− 0.07, 0.18)	0.10 (− 0.03, 0.23)	0.22	0.03 (− 0.02, 0.07)
‘Traditional’ dietary pattern						
FEV_1_						
Model 1	0.00	0.06 (− 0.07, 0.20)	0.13 (− 0.01, 0.27)	0.03 (− 0.11, 0.17)	0.54	0.01 (− 0.04, 0.06)
Model 2	0.00	0.06 (− 0.07, 0.20)	0.12 (− 0.01, 0.26)	0.02 (− 0.12, 0.16)	0.58	0.01 (− 0.04, 0.07)
FVC						
Model 1	0.00	0.04 (− 0.09, 0.17)	0.09 (− 0.04, 0.22)	0.03 (− 0.11, 0.16)	0.58	0.02 (− 0.03, 0.07)
Model 2	0.00	0.04 (− 0.09, 0.17)	0.09 (− 0.04, 0.22)	0.02 (− 0.12, 0.16)	0.64	0.02 (− 0.04, 0.07)
FEV_1_/FVC ratio						
Model 1	0.00	− 0.01 (− 0.12, 0.11)	− 0.00 (− 0.12, 0.11)	− 0.04 (− 0.16, 0.08)	0.55	− 0.02 (− 0.06, 0.03)
Model 2	0.00	− 0.02 (− 0.14, 0.09)	− 0.02 (− 0.13, 0.10)	− 0.04 (− 0.16, 0.08)	0.53	− 0.02 (− 0.06, 0.03)
FEF_25–75_						
Model 1	0.00	0.04 (− 0.08, 0.16)	0.05 (− 0.07, 0.16)	− 0.03 (− 0.15, 0.09)	0.68	− 0.02 (− 0.06, 0.03)
Model 2	0.00	0.03 (− 0.08, 0.15)	0.03 (− 0.08, 0.15)	− 0.04 (− 0.16, 0.09)	0.59	− 0.02 (− 0.06, 0.03)
‘Processed’ dietary pattern						
FEV_1_						
Model 1	0.00	0.04 (− 0.09, 0.17)	− 0.04 (− 0.18, 0.09)	− 0.14 (− 0.29, 0.02)	0.07	− 0.04 (− 0.11, 0.02)
Model 2	0.00	0.04 (− 0.09, 0.18)	− 0.04 (− 0.18, 0.10)	− 0.12 (− 0.29, 0.04)	0.11	− 0.04 (− 0.11, 0.03)
FVC						
Model 1	0.00	0.01 (− 0.11, 0.14)	− 0.06 (− 0.19, 0.07)	− 0.18 (− 0.33, − 0.03)	0.02	− 0.05 (− 0.12, 0.01)
Model 5	0.00	0.02 (− 0.11, 0.14)	− 0.05 (− 0.19, 0.08)	− 0.16 (− 0.32, − 0.00)	0.04	− 0.05 (− 0.12, 0.01)
FEV_1_/FVC ratio						
Model 1	0.00	0.02 (− 0.09, 0.13)	0.05 (− 0.07, 0.17)	0.11 (− 0.03, 0.24)	0.12	0.03 (− 0.02, 0.09)
Model 2	0.00	0.01 (− 0.10, 0.12)	0.06 (− 0.06, 0.18)	0.11 (− 0.03, 0.25)	0.11	0.04 (− 0.02, 0.10)
FEF_25–75_						
Model 1	0.00	0.01 (− 0.10, 0.13)	− 0.03 (− 0.15, 0.09)	− 0.06 (− 0.20, 0.07)	0.30	− 0.04 (− 0.09, 0.02)
Model 2	0.00	0.02 (− 0.10, 0.13)	− 0.01 (− 0.13, 0.11)	− 0.04 (− 0.18, 0.11)	0.58	− 0.02 (− 0.08, 0.04)

The number of participants included in the analysis was 2950 for FEV_1_ and 3085 for both FVC and FEF_25–75_

FEV_1_: forced expiratory volume in 1 s; FVC: forced vital capacity; FEF_25–75_: forced expiratory flow at 25–75% of FVC

Multivariable model 1: sex and total energy intake

Multivariable model 2: further adjusted for maternal education, housing tenure at birth, financial difficulty during pregnancy, maternal ethnicity, index of multiple deprivation, rural living location, maternal and paternal history of atopic disease, older sibling, younger sibling, breastfeeding, maternal smoking, vigorous physical activity, history of food allergy, and season when the FFQ was completed

^a^Linear trend was tested by treating the median values of quartiles as a continuous variable

There were similar associations with pre-bronchodilator lung function measures (Additional file 1: Table S6) although generally weaker. The positive associations between the ‘health-conscious’ dietary pattern and FEV_1_ and FVC were robust to further adjustment for the ‘processed’ and ‘traditional’ dietary patterns, maternal ‘health-conscious’ dietary pattern in pregnancy, and any supplement use, or exclusion of children of non-white mothers (~ 2.6%), and those with extreme energy intakes (Additional file 1: Table S7). The association between the ‘processed’ dietary pattern and FVC was not materially attenuated in these sensitivity analyses. We also found similar findings when inverse probability weighting was applied to correct for potential selection bias due to loss-to-follow-up (data not shown). There was the same positive association with FVC at 8 years for the ‘health-conscious’ pattern score (Additional file 1: Table S8). The association with FVC at 15.5 years was attenuated when adjusted for FVC at 8 years (regression coefficient for top versus bottom quartile 0.10, 95% CI − 0.04 to 0.25, P-trend 0.17); but there was a strong association when measurements at both time points were considered in a multilevel model (0.16, 95% CI 0.08 to 0.24, P-trend < 0.001).

The positive associations with the ‘health-conscious’ dietary pattern were not explained by dietary TAC, intake of antioxidant vitamins (vitamin C, vitamin E, carotene) and trace elements (zinc and selenium), vitamin D, preformed vitamin A, long-chain omega-3 fatty acids from fish, or fibre, nor by BMI or FMI at 9 years of age when we further adjusted for these factors separately (Additional file 1: Table S9). Similarly, the associations with the ‘processed’ dietary pattern could not be explained by these potential mediators, although adjustment for zinc intake partially attenuated the negative association with FVC (regression coefficient comparing top versus bottom quartiles − 0.11, 95% CI − 0.28 to 0.06, P for trend 0.17).

We did not find clear evidence of an interaction between dietary patterns and sex or PM_10_ on lung function measures. However, there was evidence of effect modification by asthma status at 7.5 years; in children with asthma the ‘processed’ pattern was negatively associated with FEV_1_ (regression coefficient per SD − 0.20, 95% CI − 0.39 to − 0.00) and FVC (− 0.21, 95% CI − 0.40 to − 0.02), but not in children without asthma (− 0.00, 95% CI − 0.08 to 0.07, and vs − 0.02, 95% CI − 0.09 to 0.06, respectively; P for interaction 0.02 for both outcomes) (Additional file 1: Table S10). These inverse associations were not materially changed after further adjustment for frequency of asthma treatment (data not shown). We found weak evidence of effect modification by maternal smoking in childhood; the associations between the ‘health-conscious’ pattern and FEV_1_ and FEF_25-75_ were much stronger in children of mothers who smoked ≥ 15 cigarettes/day (Additional file 1: Table S11). There was no effect modification by the polymorphic variants of most antioxidant genes (*GSTM1, GSTT1, GSTP1, and AHR*). However, the associations between the ‘health-conscious’ pattern and lung function were modified by *GPX4* rs713041 genotype; positive associations were suggested in carriers of the T allele, but not in those who were homozygous for the C allele (Additional file 1: Table S12). Post hoc, when we combined homozygous and heterozygous carriers of the T allele, a strong interaction was observed (Table 3). In stratified analysis by different genotypes of the *SCGB1A1* rs3741240 SNP, there were positive associations between the ‘health-conscious’ pattern and FEV_1_, FVC, and FEF_25-75_ only in homozygous carriers of the G allele (P for interaction 0.007, 0.005, and 0.08, respectively); similarly, the ‘processed’ pattern was negatively associated with these outcomes only in the GG group (Table 4). We found no other evidence of effect modification.

**Table 3:** Linear regression coefficients (95% confidence interval) for post-bronchodilator lung function measures (z scores) at 15.5 years according to quartiles of the ‘health-conscious’ dietary pattern score at 7 years, stratified by rs713041 polymorphism genotypes of *GPX4* gene

	Quartiles of dietary pattern score				P for	P for
	Q1	Q2	Q3	Q4	trend^a^	interaction
‘Health-conscious’ dietary pattern						
FEV_1_						
Genotype CC	0.00	0.12 (− 0.16, 0.40)	− 0.03 (− 0.32, 0.25)	− 0.04 (− 0.34, 0.26)	0.54	
Genotype CT/TT	0.00	0.10 (− 0.09, 0.30)	0.13 (− 0.07, 0.33)	0.25 (0.04, 0.46)	0.02	0.009
FVC						
Genotype CC	0.00	0.12 (− 0.15, 0.38)	0.02 (− 0.26, 0.29)	0.05 (− 0.24, 0.33)	0.97	
Genotype CT/TT	0.00	0.12 (− 0.06, 0.31)	0.09 (− 0.10, 0.28)	0.26 (0.06, 0.47)	0.02	0.03
FEV_1_/FVC ratio						
Genotype CC	0.00	0.05 (− 0.18, 0.28)	− 0.05 (− 0.29, 0.19)	− 0.15 (− 0.40, 0.11)	0.15	
Genotype CT/TT	0.00	− 0.06 (− 0.23, 0.11)	0.05 (− 0.12, 0.21)	− 0.03 (− 0.21, 0.14)	0.92	0.38
FEF_25–75_						
Genotype CC	0.00	0.05 (− 0.18, 0.28)	− 0.09 (− 0.33, 0.15)	− 0.21 (− 0.46, 0.04)	0.04	
Genotype CT/TT	0.00	0.14 (− 0.03, 0.31)	0.12 (− 0.05, 0.29)	0.21 (0.02, 0.39)	0.05	0.001

Multivariable model: sex, total energy intake, maternal education, housing tenure at birth, financial difficulty during pregnancy, maternal ethnicity, index of multiple deprivation, rural living location, maternal and paternal history of atopic disease, older sibling, younger sibling, breastfeeding, maternal smoking, vigorous physical activity, history of food allergy, and season when the FFQ was completed

FEV_1_: forced expiratory volume in 1 s; FVC: forced vital capacity; FEF_25–75_: forced expiratory flow at 25–75% of FVC; *GPX4*: glutathione peroxidase 4

^a^Linear trend was tested by treating the median values of quartiles as a continuous variable

In CC and CT/TT groups of *GPX4*-rs713041, sample sizes were 747 and 1598 for FEV_1_, and 778 and 1679 for both FVC and FEF_25-75_, respectively

**Table 4:** Linear regression coefficients (95% confidence interval) for post-bronchodilator lung function measures (z scores) at 15.5 years according to quartiles of dietary pattern score at 7 years, stratified by rs3741240 polymorphism genotypes of *SCGB1A1* gene

	Quartiles of dietary pattern score				P for	P for
	Q1	Q2	Q3	Q4	trend^a^	interaction
‘Health-conscious’ dietary pattern						
FEV_1_						
Genotype GG	0.00	0.20 (− 0.04, 0.44)	0.30 (0.05, 0.55)	0.48 (0.21, 0.74)	< 0.001	
Genotype GA	0.00	0.00 (− 0.23, 0.23)	− 0.01 (− 0.24, 0.22)	0.08 (− 0.16, 0.32)	0.47	0.02
Genotype AA	0.00	0.30 (− 0.13, 0.72)	0.05 (− 0.36, 0.47)	− 0.11 (− 0.54, 0.33)	0.32	0.007
FVC						
Genotype GG	0.00	0.15 (− 0.08, 0.37)	0.25 (0.02, 0.49)	0.47 (0.22, 0.72)	< 0.001	
Genotype GA	0.00	0.08 (− 0.14, 0.29)	0.05 (− 0.16, 0.27)	0.16 (− 0.07, 0.39)	0.19	0.06
Genotype AA	0.00	0.21 (− 0.20, 0.63)	0.05 (− 0.35, 0.46)	− 0.12 (− 0.55, 0.31)	0.37	0.005
FEV_1_/FVC ratio						
Genotype GG	0.00	0.06 (− 0.14, 0.25)	0.00 (− 0.20, 0.21)	− 0.03 (− 0.24, 0.19)	0.64	
Genotype GA	0.00	− 0.06 (− 0.27, 0.14)	− 0.07 (− 0.28, 0.13)	− 0.10 (− 0.32, 0.12)	0.41	0.60
Genotype AA	0.00	0.00 (− 0.35, 0.35)	− 0.10 (− 0.45, 0.24)	− 0.10 (− 0.46, 0.26)	0.52	0.94
FEF_25–75_						
Genotype GG	0.00	0.21 (0.00, 0.41)	0.22 (0.01, 0.43)	0.30 (0.07, 0.53)	0.02	
Genotype GA	0.00	0.01 (− 0.19, 0.20)	− 0.06 (− 0.25, 0.14)	0.01 (− 0.20, 0.22)	0.98	0.08
Genotype AA	0.00	0.02 (− 0.35, 0.39)	− 0.09 (− 0.45, 0.27)	− 0.15 (− 0.53, 0.24)	0.36	0.08
‘Processed’ dietary pattern						
FEV_1_						
Genotype GG	0.00	− 0.20 (− 0.43, 0.03)	− 0.15 (− 0.40, 0.10)	− 0.41 (− 0.69, − 0.13)	0.008	
Genotype GA	0.00	0.19 (− 0.02, 0.40)	− 0.01 (− 0.23, 0.22)	− 0.09 (− 0.36, 0.18)	0.36	0.23
Genotype AA	0.00	0.29 (− 0.11, 0.68)	0.42 (0.00, 0.85)	0.22 (− 0.30, 0.74)	0.27	0.07
FVC						
Genotype GG	0.00	− 0.24 (− 0.46, − 0.02)	− 0.13 (− 0.37, 0.11)	− 0.32 (− 0.59, − 0.05)	0.04	
Genotype GA	0.00	0.22 (0.02, 0.42)	− 0.03 (− 0.25, 0.18)	− 0.18 (− 0.43, 0.08)	0.09	0.79
Genotype AA	0.00	0.26 (− 0.13, 0.65)	0.42 (0.01, 0.83)	0.16 (− 0.34, 0.67)	0.34	0.13
FEV_1_/FVC ratio						
Genotype GG	0.00	0.04 (− 0.14, 0.23)	− 0.04 (− 0.25, 0.16)	− 0.03 (− 0.26, 0.20)	0.67	
Genotype GA	0.00	− 0.10 (− 0.29, 0.09)	0.09 (− 0.11, 0.29)	0.10 (− 0.14, 0.34)	0.24	0.14
Genotype AA	0.00	0.04 (− 0.29, 0.37)	0.02 (− 0.33, 0.38)	0.19 (− 0.24, 0.62)	0.44	0.71
FEF_25–75_						
Genotype GG	0.00	− 0.13 (− 0.33, 0.06)	− 0.21 (− 0.42, 0.00)	− 0.30 (− 0.54, − 0.06)	0.01	
Genotype GA	0.00	0.08 (− 0.10, 0.26)	0.07 (− 0.12, 0.26)	0.01 (− 0.22, 0.24)	0.91	0.04
Genotype AA	0.00	0.16 (− 0.19, 0.50)	0.22 (− 0.15, 0.58)	0.13 (− 0.33, 0.58)	0.49	0.20

Multivariable model: sex, total energy intake, maternal education, housing tenure at birth, financial difficulty during pregnancy, maternal ethnicity, index of multiple deprivation, rural living location, maternal and paternal history of atopic disease, older sibling, younger sibling, breastfeeding, maternal smoking, vigorous physical activity, history of food allergy, and season when the FFQ was completed

FEV_1_: forced expiratory volume in 1 s; FVC: forced vital capacity; FEF_25–75_: forced expiratory flow at 25–75% of FVC; *SCGB1A1*: Secretoglobin family 1A member 1

^a^Linear trend was tested by treating the median values of quartiles as a continuous variable

In GG, GA, and AA groups of *SCGB1A1*-rs3741240, sample sizes were 1063, 1124, and 342 for FEV_1_, and 1115, 1173, and 354 for both FVC and FEF_25-75_, respectively

### Incident asthma

We did not find evidence of any association between the three dietary pattern scores and incident asthma (Table 5), and this was not changed after we carried out various sensitivity analyses (Additional file 1: Table S13). We also did not find evidence of effect modification by sex, PM_10_, maternal smoking, or polymorphic variants of most antioxidant genes (*GSTM1, GSTT1, AHR, GPX4,* and *SCGB1A1*). The only exception was a weak interaction between the ‘traditional’ dietary pattern and the *GSTP1* rs1695 SNP (odds ratio per SD 1.90, 95% CI 1.16 to 3.13, in GG group vs 0.95, 95% CI 0.78 to 1.17, in GA group and 1.00, 95% CI 0.80 to 1.24, in AA group; P for interaction 0.04).

**Table 5:** Odds ratio (95% confidence interval) for incident asthma at 11 or 14 years according to quartiles of dietary pattern score at 7 years, adjusted for potential confounders

	Quartiles of dietary pattern score				P for trend^a^	Per SD
	Q1	Q2	Q3	Q4		
‘Health-conscious’ dietary pattern						
Cases/non-cases	86/984	94/1043	102/1081	109/1051		
Model 1	1.00	1.02 (0.75–1.38)	1.05 (0.78–1.43)	1.10 (0.80–1.50)	0.52	1.03 (0.92, 1.15)
Model 2	1.00	0.99 (0.73–1.36)	1.03 (0.75–1.41)	1.04 (0.74–1.45)	0.80	1.00 (0.89, 1.13)
‘Traditional’ dietary pattern						
Cases/non-cases	90/1085	101/1040	103/1009	97/1025		
Model 1	1.00	1.16 (0.86–1.56)	1.21 (0.90–1.64)	1.06 (0.77–1.45)	0.67	1.01 (0.90, 1.13)
Model 2	1.00	1.20 (0.89–1.63)	1.24 (0.91–1.69)	1.11 (0.80–1.54)	0.49	1.02 (0.91, 1.15)
‘Processed’ dietary pattern						
Cases/non-cases	107/1126	104/1073	92/1028	88/932		
Model 1	1.00	0.98 (0.73–1.30)	0.89 (0.66–1.21)	0.86 (0.61–1.21)	0.33	1.00 (0.87, 1.14)
Model 2	1.00	0.99 (0.74–1.33)	0.94 (0.68–1.29)	0.88 (0.61–1.27)	0.46	1.01 (0.87, 1.17)

The number of participants included in the analysis was 4491

Multivariable model 1: sex and total energy intake

Multivariable model 2: further adjusted for maternal education, housing tenure at birth, financial difficulty during pregnancy, maternal ethnicity, index of multiple deprivation, rural living location, maternal and paternal history of atopic disease, older sibling, younger sibling, breastfeeding, maternal smoking, vigorous physical activity, history of food allergy, and season when the FFQ was completed

^a^Linear trend was tested by treating the median values of quartiles as a continuous variable

## Discussion

In ALSPAC children, we found that a higher ‘health-conscious’ dietary pattern score in mid-childhood was associated with a higher subsequent FVC and FEV_1_, whereas a higher ‘processed’ pattern score was associated with a lower FVC and FEV_1_, independent of multiple SES indicators. We did not find evidence for an association between the ‘traditional’ pattern score and lung function, or between any dietary pattern and incident asthma. To our knowledge, these are novel findings, robust to various sensitivity analyses.

The differences in FEV_1_ between the top and bottom quartiles of ‘health-conscious’ and ‘processed’ dietary pattern scores were comparable to the mean difference in z-scores of pre-bronchodilator FEV_1_ comparing children with and without asthma (0.24, 95% CI 0.11, 0.37) [29]; thus were clinically important. Associations were similar for FEV_1_ and FVC (hence no association with their ratio), suggesting a proportional influence on airway and alveolar development. Furthermore, the stronger associations with post- than pre-bronchodilator FEV_1_ suggest that dietary patterns may be more related to growth and calibre, rather than tone, of large and small airways. Given that dietary patterns [22] and lung function [42] track during childhood, the association observed between a healthier dietary pattern at 7 years and higher FVC at 8 years would suggest a beneficial effect earlier in lung development. However, the residual effect estimates for the association between the ‘health-conscious’ pattern and FVC at 15.5 years after adjusting for FVC at 8 years, suggests that it may be having an additional effect on lung function growth between these two ages. To our knowledge, this study is the first to show prospective associations between empirically derived dietary patterns in mid-childhood and lung function measures in the general population.

### Potential mechanisms

Dietary antioxidants have received attention for decades because of their anti-inflammatory effects and the transition from a traditional to a modern diet [8]. Despite the strong link between dietary patterns and antioxidant intake in our study, their associations with lung function measures were not explained by dietary antioxidants, whether considered individually (vitamins C and E or β-carotene) or collectively (TAC). In the Swedish birth cohort, BAMSE, with a similar timeline to this study for exposure and outcome assessments, higher dietary TAC was associated with lower risk of allergic asthma [43] and, in those with asthma, a higher FEV_1_ [44]. In contrast, for the antioxidant-rich ‘health-conscious’ pattern, we did not find evidence of association with incident asthma, nor evidence of effect modification on associations with lung function by asthma at baseline. Furthermore, we found no evidence of effect modification by common polymorphisms of antioxidant genes (*GSTM1, GSTT1, GSTP1, and AHR*) or by air pollution (PM_10_, an indicator of environmental oxidants). These findings suggest that the potential benefits of the ‘health-conscious’ pattern on lung function may not be explained by higher antioxidant intake. On the other hand, we found evidence of interactions between the ‘health-conscious’ pattern and maternal smoking and another antioxidant gene variant on lung function. The positive associations between the ‘health conscious’ pattern and lung function were only seen in carriers of the T allele of the rs713041 polymorphism of the *GPX4* gene, who have an impaired antioxidant defence because of weaker GPX enzyme function, particularly when selenium intake is suboptimal [34]. Thus, these individuals might be expected to derive the greatest benefit from a higher intake of selenium. Whilst there was a positive correlation between the ‘health-conscious’ pattern score and selenium intake in this study, adjustment for selenium did not materially change these findings. However, accurate estimation of selenium intake is difficult using dietary questionnaires because of the limitations of food composition data in capturing large regional and seasonal variations [45], and in differentiating organic from inorganic selenium [46].

We also found evidence for interactions between both ‘health-conscious’ and ‘processed’ patterns and the *SCGB1A1* rs3741240 SNP which regulates circulating levels of CC16 protein [37]. CC16 is an anti-inflammatory pneumoprotein produced by club cells in the airways, and its lower concentrations have been associated with lung function deficits [18, 47], increased airway resistance, and hyperresponsiveness [47]. In this study, the ‘health-conscious’ pattern was associated with better lung function only in children with a genetic tendency to produce more CC16 (GG genotype). We have recently reported a strong positive association between preformed vitamin A intake and FEV_1_ and FEF_25-75_ in childhood, with a similar interaction with the *SCGB1A1* polymorphism indicating a greater potential for up-regulation in the GG group [29]. However, findings in this study were not materially changed after adjusting for preformed vitamin A intake. We therefore cannot explain the interactions we observed with the *SCGB1A1* polymorphism.

The ‘health-conscious’ and ‘processed’ patterns generally describe a nutrient-rich, versus an energy-dense, nutrient-poor diet, respectively [22, 31]. In this study, we explored a long list of potential mediators that have been postulated to explain the link between nutrition and respiratory outcomes, including dietary fibre, vitamin D, long-chain omega-3, and indicators of adiposity [48]. Although the ‘health-conscious’ and ‘processed’ patterns were strongly correlated with most of these factors (in opposite directions), the associations with lung function measures were not explained by them individually. On the one hand, this emphasises the importance of a ‘whole diet’ approach, which can go beyond individual nutrients, particularly those that have been implicated as potential risk factors to date. On the other hand, this underscores the need for expanding the search for other underlying mechanisms. We did however find that zinc intake partially explained the negative association between the ‘processed’ pattern and lung function. In the same cohort, we have previously found that lower maternal intake of zinc in pregnancy was associated with lower childhood lung function [49].

Of note, as the ‘health-conscious’ and ‘processed’ patterns scores were derived not to be correlated, their opposite associations with lung function may reflect different mechanisms. We speculate that the link between diet and lung function may be mediated by altered microbiota [50] or altered DNA methylation [51, 52].

### Strengths and limitations

The post-bronchodilator assessment of lung function, provided by the ALSPAC cohort, enabled us to better assess airway growth by eliminating reversible airflow limitation. Also, the availability of genotype data enabled us to test gene-diet interactions. We controlled for numerous potential confounders in the analyses; however, the possibility of unmeasured or residual confounding cannot be ruled out. Regarding the multiple analyses carried out, given the a priori nature of the hypotheses and the correlation between lung function measures, it did not seem appropriate to correct for multiple testing [53]; however, the risk of false-positive findings should be considered in the interpretation of our findings, particularly the exploratory analyses including gene-diet interactions. A sizable proportion of eligible children at 7 years were not included in our analyses, but our inverse probability weighting analysis showed that this is unlikely to have biased our findings, as generally expected in longitudinal studies [54]. However, as it is evident that SES influenced both recruitments [19, 20] and dropouts [55] in ALSPAC, we cannot rule out the possibility that collider bias (M bias) may have influenced the results if a factor linked to participation also influences lung function. Whilst misclassification of the dietary exposures was inevitable, the prospective nature of the study makes it more likely that such misclassification would have been non-differential with respect to the outcomes. The empirical method for identifying dietary patterns has its limitations as it involves important but arbitrary decisions including the consolidation of food items into groups, the number of components to extract, and the labelling of the components [4]. Evidence of tracking in childhood diet in this cohort, which was particularly strong for the ‘health-conscious’ and ‘processed’ patterns [22], demonstrates the reproducibility of these patterns and is reassuring. However, as the empirically derived patterns are inherently population-specific, their replication is not practically straightforward, which has implications for the generalizability of our findings to other populations. Finally, ALSPAC participants were mainly White which may also limit generalizability to other ethnicities.

## Conclusions

A healthier diet in mid-childhood was associated with higher subsequent lung function, while a diet high in processed food was associated with lower lung function. Future studies are needed to replicate our findings and to elucidate underlying mechanisms.

## Supplementary Information

**Additional** **file** **1:** **Figure** **S1.** Study profile. **Figure** **S2.** Directed acyclic graph to study covariates and potential structural confounding bias for the association between child’s dietary patterns and lung function. **Table** **S1.** Summary of the factor loadings of foods in the dietary patterns extracted using principal component analysis from the FFQ at 7 years (loadings above 0.3 are shown in bold). **Table** **S2.** Details of selected polymorphisms. **Table** **S3.** Participant characteristics according to quartiles of ‘health-conscious’ dietary pattern score at 7 years of age. **Table** **S4.** Participant characteristics according to quartiles of ‘traditional’ dietary pattern score at 7 years of age. **Table** **S5.** Participant characteristics according to quartiles of ‘processed’ dietary pattern score at 7 years of age. **Table** **S6.** Linear regression coefficients (95% confidence interval) for pre-bronchodilator lung function measures (z scores) at 15.5 years according to quartiles of dietary pattern scores at 7 years, adjusted for potential confounders. **Table** **S7.** Linear regression coefficients (95% confidence interval) for post-bronchodilator lung function measures (z scores) at 15.5 years according to quartiles of dietary pattern scores at 7 years, adjusted for additional potential confounders as well as exclusions (sensitivity analyses). **Table** **S8.** Linear regression coefficients (95% confidence interval) for pre-bronchodilator lung function measures (z scores) at 8 years according to quartiles of dietary pattern scores at 7 years, adjusted for potential confounders. **Table** **S9.** Linear regression coefficients (95% confidence interval) for post-bronchodilator lung function measures (z scores) at 15.5 years according to quartiles of dietary pattern score at 7 years, further adjusted for potential mediators. **Table** **S10.** according to quartiles of dietary pattern score at 7 years, stratified by asthma status at 7.5 years. **Table** **S11.** According to quartiles of dietary pattern score at 7 years, stratified by maternal smoking in childhood. **Table** **S12.** according to quartiles of the health-conscious dietary pattern score at 7 years, stratified by *GPX4* genotype (rs713041). **Table** **S13.** Odds ratio (95% confidence interval) for incident asthma at 11 or 14 years according to quartiles of dietary pattern score at 7 years, adjusted for additional factors as well as exclusions (sensitivity analysis).

## Data Availability

The informed consent obtained from ALSPAC participants does not allow the data to be made freely available through any third party maintained public repository. However, data used for this submission can be made available on request to the ALSPAC Executive. The ALSPAC data management plan describes in detail the policy regarding data sharing, which is through a system of managed open access. Full instructions for applying for data access can be found here: http://www.bristol.ac.uk/alspac/researchers/access/. The ALSPAC study website contains details of all the data that are available (http://www.bristol.ac.uk/alspac/researchers/our-data/).
