# Supplementary material for: Dietary patterns, lung function and asthma in childhood: a longitudinal study
Source: Respir Res. 2023 Mar 16;24:82. doi: 10.1186/s12931-023-02383-9 (PMC10022039; doi:10.1186/s12931-023-02383-9)
Supplement: Supplementary file 1 — Additional file 1: Figure S1. Study profile. Figure S2. Directed acyclic graph to study covariates and potential structural confounding bias for the association between child’s dietary patterns and lung function. Table S1. Summary of the factor loadings of foods in the dietary patterns extracted using principal component analysis from the FFQ at 7 years (loadings above 0.3 are shown in bold). Table S2. Details of selected polymorphisms. Table S3. Participant characteristics according to quartiles of ‘health-conscious’ dietary pattern score at 7 years of age. Table S4. Participant characteristics according to quartiles of ‘traditional’ dietary pattern score at 7 years of age. Table S5. Participant characteristics according to quartiles of ‘processed’ dietary pattern score at 7 years of age. Table S6. Linear regression coefficients (95% confidence interval) for pre-bronchodilator lung function measures (z scores) at 15.5 years according to quartiles of dietary pattern scores at 7 years, adjusted for potential confounders. Table S7. Linear regression coefficients (95% confidence interval) for post-bronchodilator lung function measures (z scores) at 15.5 years according to quartiles of dietary pattern scores at 7 years, adjusted for additional potential confounders as well as exclusions (sensitivity analyses). Table S8. Linear regression coefficients (95% confidence interval) for pre-bronchodilator lung function measures (z scores) at 8 years according to quartiles of dietary pattern scores at 7 years, adjusted for potential confounders. Table S9. Linear regression coefficients (95% confidence interval) for post-bronchodilator lung function measures (z scores) at 15.5 years according to quartiles of dietary pattern score at 7 years, further adjusted for potential mediators. Table S10. according to quartiles of dietary pattern score at 7 years, stratified by asthma status at 7.5 years. Table S11. According to quartiles of dietary pattern score at 7 years, stratified by m [file 12931_2023_2383_MOESM1_ESM.pdf]

Online supplementary materials

Dietary patterns, lung function and asthma in childhood: A longitudinal study

Mohammad Talaei, Pauline M. Emmett, Raquel Granell, Hossein Tabatabaeian, Kate Northstone, Anna Bergström, Seif O. Shaheen

Corresponding author:

Dr Mohammad Talaei, Wolfson Institute of Population Health, Barts and The London School of Medicine and Dentistry, Charterhouse Square, London EC1M 6B, UK. E-mail: m.talaei@qmul.ac.uk; Tel: +44(0)20 7882 2499

Contents

Online supplementary materials ... **1**
Further details ... **3**
Information on dietary patterns ... 3
Information on covariates ... 4
Genotyping ... 5
Multivariable models ... 6
Sensitivity analyses ... 7
Restricted cubic spline analysis ... 7
Inverse probability weighting ... 7
References ... **9**
Supplementary tables and figures ... **11**
Figure S1. Study profile. ... 11
Figure S2. Directed acyclic graph to study covariates and potential structural confounding bias for the association between dietary patterns and lung function in childhood. ... 12
Table S1. Summary of the factor loadings of foods in the dietary patterns extracted using principal component analysis from the FFQ at 7 years (loadings above 0.3 are shown in bold). ... 13
Table S2. Details of selected polymorphisms ... 14
Table S3: Participant characteristics according to quartiles of 'health-conscious' dietary pattern score at 7 years of age ... 15
Table S4: Participant characteristics according to quartiles of 'traditional' dietary pattern score at 7 years of age ... 17
Table S5: Participant characteristics according to quartiles of 'processed' dietary pattern score at 7 years of age ... 19
Table S6: Linear regression coefficients (95% confidence interval) for pre-bronchodilator lung function measures (z scores) at 15.5 years according to quartiles of dietary pattern scores at 7 years, adjusted for potential confounders ... 21
Table S7: Linear regression coefficients (95% confidence interval) for post-bronchodilator lung function measures (z scores) at 15.5 years according to quartiles of dietary pattern scores at 7 years, adjusted for additional potential confounders as well as exclusions (sensitivity analyses) ... 23
Table S8: Linear regression coefficients (95% confidence interval) for pre-bronchodilator lung function measures (z scores) at 8 years according to quartiles of dietary pattern scores at 7 years, adjusted for potential confounders ... 25
Table S9: Linear regression coefficients (95% confidence interval) for post-bronchodilator lung function measures (z scores) at 15.5 years according to quartiles of dietary pattern score at 7 years, further adjusted for potential mediators ... 27
Table S10: Linear regression coefficients (95% confidence interval) for post-bronchodilator lung function measures (z scores) at 15.5 years according to quartiles of dietary pattern score at 7 years, stratified by asthma status at 7.5 years ... 29
Table S11: Linear regression coefficients (95% confidence interval) for post-bronchodilator lung function measures (z scores) at 15.5 years according to quartiles of dietary pattern score at 7 years, stratified by maternal smoking in childhood ... 31
Table S12: Linear regression coefficients (95% confidence interval) for post-bronchodilator lung function measures (z scores) at 15.5 years according to quartiles of the health-conscious dietary pattern score at 7 years, stratified by *GPX4* genotype (rs713041) ... 33
Table S13: Odds ratio (95% confidence interval) for incident asthma at 11 or 14 years according to quartiles of dietary pattern score at 7 years, adjusted for additional factors as well as exclusions (sensitivity analysis) ... 34

Further details

Information on dietary patterns

The FFQ included questions about the usual consumption of 56 food groups and 12 drinks, with five frequency options ranging from 'never or rarely' to 'more than once a day' and daily consumption of specific types of bread, fat spreads/oils and milk (1). The FFQ used in ALSPAC was based on the one used by Yarnell et al. (2), which has been validated against weighed dietary records and updated in the light of a locally weighed dietary survey (3). In our unpublished findings, the comparison between results of the ALSPAC FFQ and concurrent dietary record data showed correlations of around 0.4 between most nutrient densities (adjusted for energy) estimated by 3-day dietary records and by FFQ which is comparable to those of other surveys. There were much higher correlations for some essential items, such as vegetable intake that are instrumental in dietary pattern detection. We found that this FFQ was able to reasonably rank subjects in this population according to dietary intake. Principle component analysis (PCA) with varimax rotation was performed on the standardised food items. The methods have been described in detail elsewhere (4, 5). Briefly, the number of components best representing the data was primarily chosen based on the scree-plot and the interpretability of the components. As intake of tea, coffee, bread, milk, and spreads was recorded on a daily scale rather than weekly, all variables were standardised by subtracting the mean and dividing by the standard deviation. Children who had more than 10 dietary items missing were excluded from the respective questionnaire. If 10 or fewer items were missing, it was assumed that the child did not consume those items and missing was replaced with zero. Of 57 food items/groups included in the PCA, those with loadings above 0.3 on a component were considered to have a strong association with that component and were deemed the most informative in describing the dietary patterns. Labels were given to each component to aid in the reporting and discussion of the results, although they do not perfectly describe each underlying pattern. For each child, a score was created for each component by multiplying the factor loadings by the corresponding standardised value for each food and summing across the food items. All component scores were approximately normally distributed, with a mean of 0 and an SD of 1. A high positive score suggests that the dietary pattern well describes the type of diet consumed by that child. Table E1 shows the factor loadings of foods in the dietary patterns extracted using PCA. For each of the three patterns, the following food groups loaded highly (6):

- 'Health Conscious': non-white bread, white bread (negative loading), cheese, vegetarian pies, meat substitutes (soya, tofu etc.), pulses, nuts, pasta, salad, fresh fruit, water, fish, and rice.
- 'Traditional': poultry, red meat, roast potatoes, potatoes (not chips), green vegetables, root vegetables, peas, sweetcorn, fish, and rice.

- ‘Processed’: white bread, biscuits, ice cream, milk-based puddings including custard, cakes/buns, coated poultry products, meat pies, sausages/burgers, pizza, chips, baked beans/tinned pasta, fizzy drinks, sweets, chocolate, and crisps.

Information on covariates

Living location was defined based on the 2001 Census urban/rural indicator identified by child’s postcode at 7 years of age. Data on the index of multiple deprivation (IMD) was similarly obtained through postcode. A maternal history of hay fever, asthma, and eczema was ascertained at 12 weeks of gestation, and any positive response was considered a maternal history of atopic disease. The paternal history of the atopic disease was defined similarly through questions asked about partners during pregnancy or early after delivery. Mothers were asked how many cigarettes they smoked per day when the child was 7 years of age. We defined childhood food allergy if there was any such report by mothers at 6 (to milk), 30, 54, or 81 months of age. Data on maternal ethnicity and indicators of socioeconomic status (maternal education, housing tenure and financial difficulty in pregnancy) were collected at various time points during pregnancy (8, 18, and 32 weeks of gestation) and at 8 weeks postpartum. The number of older and younger siblings was asked at 7 years; if data were missing, we used data on parity to calculate the number of older siblings. Data on breastfeeding by the 3rd month, and childcare by day nursery, were collected at 6 and 15 months of age, respectively. The frequency of child’s participation in vigorous physical activity (such as running, dance, gymnastics, netball, swimming, or aerobics) during the past month was asked at 8 years of age. We used information on supplement use collected at 78 months of age and defined overall use. Child atopy was defined by a positive reaction (maximum diameter of any detectable weal) to Dermatophagoides pteronyssinus, cat or grass (after subtracting positive saline reactions from histamine and allergen weals and excluding children unreactive to 1% histamine) at 7 years.

The child’s body mass index was calculated as weight (kg) divided by height squared (m^2), measured at age 7 years. BMI was missing for around 12% of participants included in these analyses. We used a forward stepwise logistic regression analysis to define a model that predicts BMI. Among potential variables initially included, 11 factors significantly contributed to the model (sex, total energy intake, vigorous physical activity, older siblings, younger siblings, any supplement use, season of data collection, maternal education, maternal history of atopy, financial difficulty during pregnancy, maternal smoking at 7 years). We applied this model to impute missing BMI using the corresponding coefficients of these factors. The mean ± SD of BMI was 16.2 ± 2.1 kg/m^2 originally and 16.2 ± 1.9 kg/m^2 after imputation. We used the same method to impute 187 (6.1%) missing BMI at 9 years; the only difference was that BMI at 7 years was also included in the prediction model. The mean ± SD of BMI at 9 years was 17.7 ± 2.9 kg/m^2 originally and 17.8 ± 2.7 kg/m^2 after imputation.

Fat mass (FM) was measured by dual-energy X-ray absorptiometry (DXA) at 9 years follow-up using the Lunar Prodigy DXA fan bean scanner (GE Medical Systems Lunar, Madison, WI, USA). FM index (FMI) was calculated as FM (kg) divided by height (m) raised to an optimum power calculated by regressing log (FM) on log (height) to remove the relation between FM and height (7). As the optimum power varied significantly by gender (boys: 6.39, girls: 5.13), FMI was calculated separately for boys and girls.

We estimated dietary total antioxidant capacity (TAC, μmol of Trolox equivalents per day) by combining estimated intakes of specific food items with information from a database of common foods analysed with the oxygen radical absorbance capacity (ORAC) method (8) on the average ORAC content (μmol Trolox equivalents/g). There were 31 food items (including 21 fruits and vegetables) with available ORAC values, while we did not include potential ORAC from supplements.

A full residential address history was constructed through the Algorithm for Generating Address-history and Exposures and was geocoded for pregnancy, infancy, and every year up to age 15 years (9). For geocoding, individuals for each exposure period must have had a valid address for a large proportion of days within each period (at least 90%-75%). Dispersion modelling was used to estimate residential air pollution exposures to particulate matter (PM) from different sources, taking into account residential mobility (10). Modelled daily estimates of PM <10 mm in aerodynamic diameter (PM_{10}) from local major road traffic were based on information about traffic flows, composition, speed, tailpipe and non-tailpipe emission rates, road widths, building heights for street canyons, and meteorological variables. Total PM_{10} emissions was obtained from the National Atmospheric Emissions Inventory comprising PM_{10} from roads, from other sources (emissions on a 1×1 km grid from other local combined sources, such as minor roads and industrial or domestic sources), anthropogenic sources outside the study area and secondary formation of PM from the UK and western Europe from the Met Office's Numerical Atmospheric dispersion Modeling Environment (NAME) III model (11), and a constant to represent local nonanthropogenic sources (e.g., wind-blown soil and dust). PM_{10} estimates were averaged for each period and each study child. We used an annual average of PM_{10} concentrations from total sources at age 7 years (concurrent with dietary data).

Genotyping

The majority of the children's DNA samples were extracted from cord blood or venous blood collected at age 7 years, with a small number extracted from venous blood collected at 43–61 months. ALSPAC children were genotyped using the Illumina HumanHap550 quad chip genotyping platforms by 23andme subcontracting the Wellcome Trust Sanger Institute, Cambridge, UK and the Laboratory Corporation of America, Burlington, NC, US. The resulting raw genome-wide data were subjected to standard quality control methods. Individuals were excluded on the basis of gender mismatches, minimal or excessive heterozygosity, disproportionate levels of individual missingness (>3%), and insufficient

sample replication (IBD < 0.8). Population stratification was assessed by multidimensional scaling analysis and compared with Hapmap II (release 22) European descent (CEU), Han Chinese, Japanese and Yoruba reference populations; all individuals with non-European ancestry were removed. SNPs with a minor allele frequency of <1%, a call rate of <95% or evidence for violations of Hardy-Weinberg equilibrium (P < 5E-7) were removed. Cryptic relatedness was measured as the proportion of identity by descent (IBD > 0.1). Related subjects that passed all other quality control thresholds were retained during subsequent phasing and imputation. 9,115 subjects and 500,527 SNPs passed these quality control filters. After removing 11,396 SNPs with genotype missingness above 1% due to poor quality and a further 321 subjects due to potential ID mismatches, imputation was performed using IMPUTE2 and the HRC reference panel (v1.1). Imputation quality was capped (in addition to minor allele frequency) at an imputation information metric score (info) greater than 0.95. This data was used to extract information on rs3741240 (*SCGB1A1*).

The glutathione S-transferase (GST) theta 1 (*GSTT1*) and mu 1 (*GSTM1*) gene deletion genotyping was performed using a real-time PCR method described previously (12). This enabled measurement of copy number variation with identification of hemizygotes in addition to classifying as gene present or absent. Three single nucleotide polymorphisms (SNPs) were typed by LGC Genomics (formerly KBiosciences, Hoddesdon, UK), using a competitive allele-specific PCR system (KASPar): a SNP in *GSTP1* [G313A, Ile105Val, rs1695 (formerly rs947894)], a SNP in *GPX4* (glutathione peroxidase 4; rs713041, at position 718), and a SNP in *AHR* (rs2066853). Table E2 shows the polymorphisms included in this analysis; rs1695 and rs3741240 were not in linkage disequilibrium (R^2= 0.0002).

Multivariable models

In the multivariable models, we first adjusted for sex and total energy intake (kJ·day−1). The second model additionally included maternal ethnicity (white, non-white) and three indicators of socioeconomic status at birth, namely, maternal education (secondary education, vocational, O level, A level, degree, and missing), housing tenure during pregnancy (mortgaged/owned, council rented, non-council rented, unknown/missing), financial difficulty during pregnancy (yes/no), IMD (quintile), living location (urban vs rural), and maternal history of atopic disease (yes/no), paternal history of atopic disease (yes/no), older sibling (yes/no), younger sibling (yes/no), breastfeeding by the 3rd month (never, stopped/non-exclusive, exclusive), maternal smoking when the child was 7 years of age (none, 1-9, 10-19, and ≥20/day), vigorous physical activity (none or less than once a week, 1-3 times a week, 4-6 times a week, and daily), history of food allergy (yes/no), and season when the FFQ was completed (winter, spring, summer, and autumn). Data on potential confounders in multivariable models were missing for 4.2% at most and included in the analyses as separate ‘missing’ categories.

Sensitivity analyses

The sensitivity of our findings to adjustment for other potential confounders was tested by additional mutual adjustment for other dietary patterns (‘health-conscious’, ‘processed’, and ‘traditional’ scores as quartiles) in one model, as well as adjusting for PCA-derived maternal dietary patterns at 32 weeks of gestation (‘vegetarian’, ‘confectionary’, ‘processed’, ‘traditional’ and ‘health conscious’ scores (6) as quartiles) in separated models. We also tested the impact of further adjustment for the use of any supplement (yes/no) and child atopy (yes/no). The associations of our exposure variables with pre-bronchodilator lung function measures were analysed too. We also explored the impact of excluding children of non-white mothers and those with an extreme total energy intake above the 95th percentile or below the 5th percentile (in separate models).

Restricted cubic spline analysis

Restricted cubic spline analysis was used to examine the shape of relationship between dietary pattern scores and lung function measures and asthma in multivariable-adjusted models. We selected the number of knots based on Akaike information criteria (AIC) values to fit the best-approximating model, chose the first knot as the reference, and tested for linearity by the Wald test.

Inverse probability weighting

Inverse probability weighting is a technique for correcting selection bias (13). In a two-step method, the probability of selection in the study is estimated for everyone based on a given set of covariates and exposure; then the inverse of this probability is included in the analysis as a weight. Inverse probability weighting creates a pseudo-population in which each selected subject accounts for those with similar characteristics who were not selected.

Accordingly, among 6,027 children with data on dietary patterns who were not diagnosed with current asthma at 7.5 years, we estimated the probability of selection of 4,491 children for given values of covariates using a logistic regression model. Unselected children were those with unknown asthma status at 7.5, 11, or 14 years. Similarly, we estimated the probability of selection of 2,950 children with data on all lung function measures at 15.5 years for given values of covariates among 8,035 children with data on dietary patterns. These covariates included all factors in model 2 (namely, sex, total energy intake, maternal education, housing tenure during pregnancy, financial difficulty during pregnancy, maternal ethnicity, maternal history of atopic disease, maternal age, maternal smoking, older sibling, younger sibling, IMD, living location, breastfeeding, vigorous physical activity, history of food allergy, and season of dietary data collection), plus quartiles of the three dietary patterns score (processed, traditional, and health conscious). Then, we assigned the inverse of this probability as the weight for each participant, and carried out a multivariable weighted logistic or linear regression analysis to test the associations of quartiles of dietary patterns with incident asthma or lung function measures in a pseudo-population,

which, in contrast to the selected population, is unaffected by selection bias due to these factors. In other words, this approach tests if the observed associations in the main analysis were sensitive to unknown asthma status at baseline (for incident asthma) or loss to follow-up (for both lung function measures and incident asthma).

References

1. Emmett P. Dietary assessment in the Avon Longitudinal Study of Parents and Children. *Eur J Clin Nutr*. 2009;63 Suppl 1:S38-44.

2. Yarnell JW, Fehily AM, Milbank JE, et al. A short dietary questionnaire for use in an epidemiological survey: comparison with weighed dietary records. *Hum Nutr Appl Nutr*. 1983;37(2):103-12.

3. Rogers I, Emmett P. Diet during pregnancy in a population of pregnant women in South West England. ALSPAC Study Team. Avon Longitudinal Study of Pregnancy and Childhood. *Eur J Clin Nutr*. 1998;52(4):246-50.

4. Northstone K, Emmett P. Multivariate analysis of diet in children at four and seven years of age and associations with socio-demographic characteristics. *Eur J Clin Nutr*. 2005;59(6):751-60.

5. Northstone K, Emmett PM. Are dietary patterns stable throughout early and mid-childhood? A birth cohort study. *Br J Nutr*. 2008;100(5):1069-76.

6. Emmett PM, Jones LR, Northstone K. Dietary patterns in the Avon Longitudinal Study of Parents and Children. *Nutr Rev*. 2015;73 Suppl 3:207-30.

7. Wells JC, Cole TJ, steam As. Adjustment of fat-free mass and fat mass for height in children aged 8 y. *Int J Obes Relat Metab Disord*. 2002;26(7):947-52.

8. Wu X, Beecher GR, Holden JM, et al. Lipophilic and hydrophilic antioxidant capacities of common foods in the United States. *J Agric Food Chem*. 2004;52(12):4026-37.

9. Boyd A, Thomas R, Hansell AL, et al. Data Resource Profile: The ALSPAC birth cohort as a platform to study the relationship of environment and health and social factors. *Int J Epidemiol*. 2019;48(4):1038-39k.

10. Gulliver J, Elliott P, Henderson J, et al. Local- and regional-scale air pollution modelling (PM10) and exposure assessment for pregnancy trimesters, infancy, and childhood to age 15years: Avon Longitudinal Study of Parents And Children (ALSPAC). *Environ Int*. 2018;113:10-19.

11. Jones A, Thomson D, Hort M, et al., editors. The U.K. Met Office's Next-Generation Atmospheric Dispersion Model, NAME III2007; Boston, MA: Springer US.

12. Rose-Zerilli MJ, Barton SJ, Henderson AJ, et al. Copy-number variation genotyping of GSTT1 and GSTM1 gene deletions by real-time PCR. *Clin Chem*. 2009;55(9):1680-5.

13. Hernan MA, Hernandez-Diaz S, Robins JM. A structural approach to selection bias. *Epidemiology*. 2004;15(5):615-25.

14. Kim DK, Cho MH, Hersh CP, et al. Genome-wide association analysis of blood biomarkers in chronic obstructive pulmonary disease. *Am J Respir Crit Care Med*. 2012;186(12):1238-47.

15. Gilliland FD, Gauderman WJ, Vora H, et al. Effects of glutathione-S-transferase M1, T1, and P1 on childhood lung function growth. *Am J Respir Crit Care Med*. 2002;166(5):710-6.

16. Piacentini S, Polimanti R, Simonelli I, et al. Glutathione S-transferase polymorphisms, asthma susceptibility and confounding variables: a meta-analysis. *Mol Biol Rep*. 2013;40(4):3299-313.

17. Shaheen SO, Rutterford CM, Lewis SJ, et al. Maternal selenium status in pregnancy, offspring glutathione peroxidase 4 genotype, and childhood asthma. *J Allergy Clin Immunol*. 2015;135(4):1083-5 e3.

18. Meplan C, Crosley LK, Nicol F, et al. Functional effects of a common single-nucleotide polymorphism (GPX4c718t) in the glutathione peroxidase 4 gene: interaction with sex. *Am J Clin Nutr*. 2008;87(4):1019-27.

19. Villette S, Kyle JA, Brown KM, et al. A novel single nucleotide polymorphism in the 3' untranslated region of human glutathione peroxidase 4 influences lipoxygenase metabolism. *Blood Cells Mol Dis*. 2002;29(2):174-8.

20. Kobayashi S, Sata F, Sasaki S, et al. Genetic association of aromatic hydrocarbon receptor (AHR) and cytochrome P450, family 1, subfamily A, polypeptide 1 (CYP1A1) polymorphisms with dioxin blood concentrations among pregnant Japanese women. *Toxicol Lett*. 2013;219(3):269-78.

Supplementary tables and figures

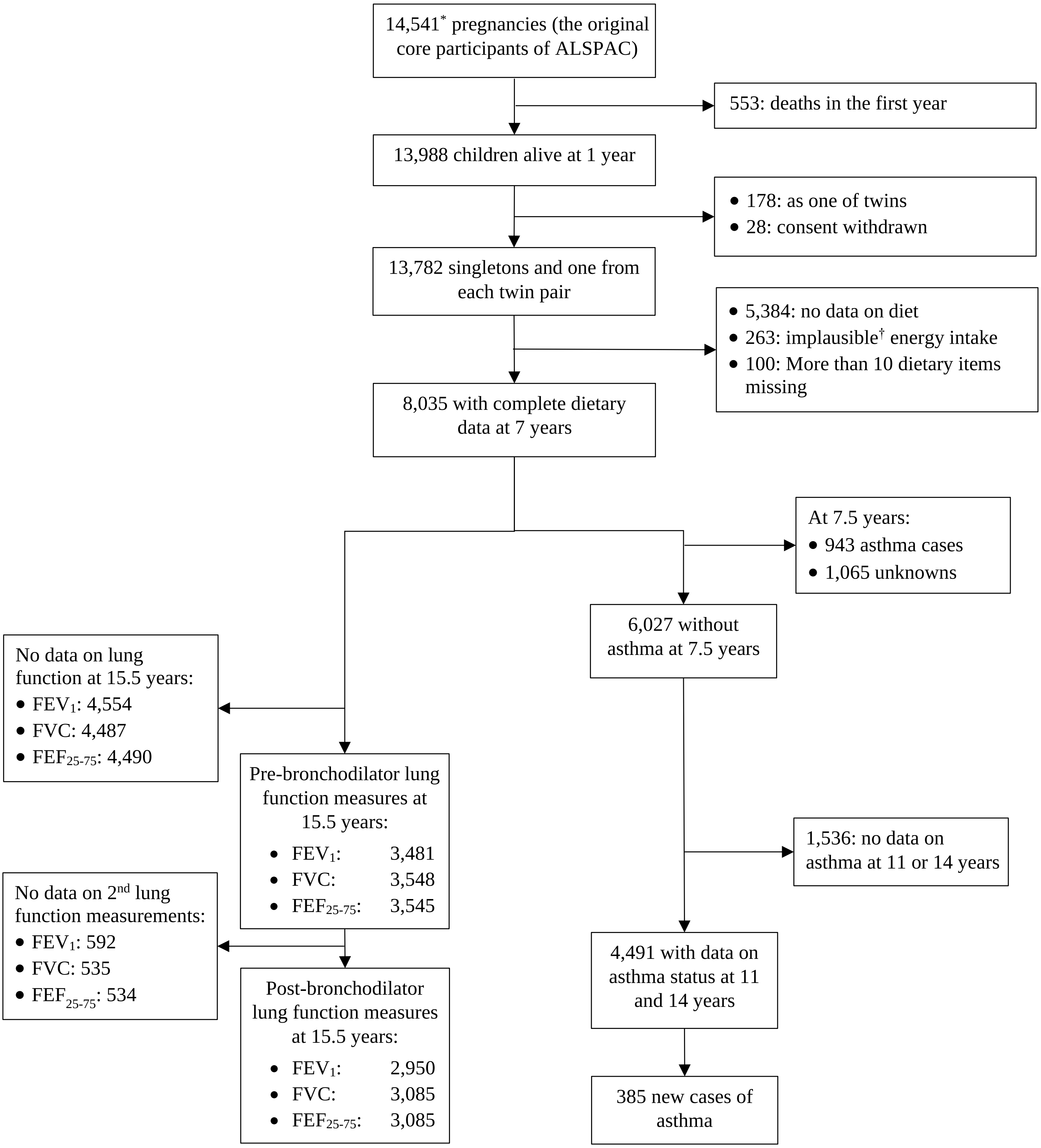

Figure S1. Study profile.

† Weekly total energy intake of <15000 kJ or >140000 kJ

* The total ALSPAC sample size for analyses using data collected after the age of seven is 15,454 pregnancies (resulting in 14,901 alive children at one year of age), but we used Phase I enrolment as information from younger ages was needed.

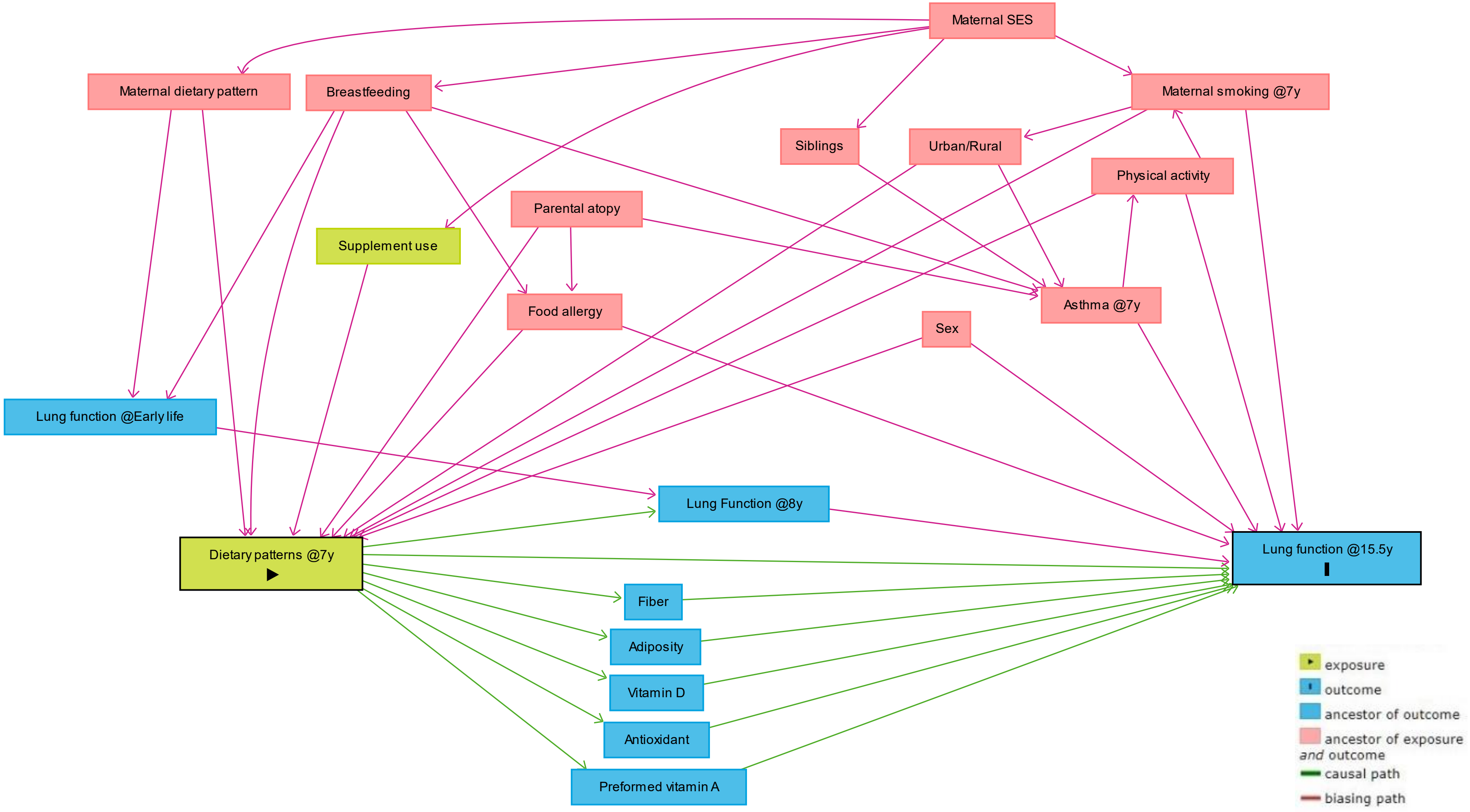

Figure S2. Directed acyclic graph to study covariates and potential structural confounding bias for the association between dietary patterns and lung function in childhood.

Table S1. Summary of the factor loadings of foods in the dietary patterns extracted using principal component analysis from the FFQ at 7 years (loadings above 0.3 are shown in bold).

Food item	'Healthy/Health Conscious'	'Traditional'	'Processed'
Variance explained	*(3.81%)*	*(7.03%)*	*(7.32%)*
White bread	**-0.339**	0.023	**0.302**
Non-white bread	**0.418**	-0.026	-0.290
Biscuits	-0.082	-0.034	**0.509**
Ice cream	0.086	0.047	**0.510**
Milk based puddings including custard	0.246	0.230	**0.367**
Other puddings	0.255	0.240	0.289
Cakes/buns	0.144	0.032	**0.346**
Poultry	-0.154	**0.627**	-0.099
Red Meat	-0.075	**0.608**	0.102
Cold meats	-0.187	0.239	0.172
Coated poultry products	-0.207	0.212	**0.387**
Meat pies	-0.075	0.131	**0.325**
Sausages, burgers	-0.161	-0.009	**0.395**
Pizza	0.234	-0.048	**0.301**
Fish	**0.319**	**0.312**	-0.071
Eggs	0.299	0.171	0.126
Cheese	**0.320**	0.085	0.052
Vegetarian pies	**0.448**	-0.114	0.095
Meat substitutes (soya, Tofu etc.)	**0.523**	0.208	-0.051
Pulses	**0.486**	-0.010	-0.110
Nuts	**0.401**	-0.089	-0.049
Chips	-0.072	-0.050	**0.511**
Roast potatoes	-0.182	**0.409**	0.245
Potatoes (not chips)	0.075	**0.448**	0.009
Pasta	**0.417**	0.197	-0.099
Rice	**0.352**	**0.324**	-0.020
Baked beans/tinned pasta	0.038	0.131	**0.377**
Green vegetables	0.255	**0.702**	-0.105
Root vegetables	0.266	**0.584**	-0.078
Peas	0.156	**0.517**	-0.014
Sweetcorn	0.270	**0.412**	-0.028
Salad	**0.421**	0.283	-0.115
Fresh fruit	**0.339**	0.296	-0.004
Fruit juice	0.251	0.061	0.041
Fizzy drinks	-0.084	0.015	**0.484**
Tea/coffee	-0.002	0.035	-0.060
Water	**0.301**	0.135	-0.110
Flavored milk drinks	0.128	0.060	0.189
Sweets	-0.013	-0.016	**0.522**
Chocolate	-0.062	-0.041	**0.516**
Crisps	-0.076	-0.068	**0.439**

Extracted from 'Dietary patterns in the Avon Longitudinal Study of Parents and Children' (6).

Table S2. Details of selected polymorphisms

Variant	Chr.	Position (GRCh38)	Gene	Location	Alleles	MAF*	Effect allele
rs3741240	11	62419070	*SCGB1A1*	5' UTR	G/A	0.35 (A)	G[†]
rs1695**	11	67585218	*GSTP1*	Coding sequence	A/G	0.34 (G)	G[††]
GSTT1 del	22	NA	*GSTT1*	Coding sequence	NA	NA	Null[§]
GSTM1 del	1	NA	*GSTM1*	Coding sequence	NA	NA	Null[§§]
rs713041	19	1106616	*GPX4*	3' UTR	C/T	0.44 (T)	T[‡]
rs2066853	7	17339486	*AHR*	Coding sequence	G/A	0.12 (A)	A[‡‡]

Chr.: Chromosome; MAF: Minor allele frequency; UTR: Untranslated region; *SCGB1A1*: Secretoglobin family 1A member 1 [Club cell secretory (CC16) protein coding gene]; *GSTP1*: Glutathione S-transferase pi 1; *GSTT1*: glutathione S-transferase theta 1; *GSTM1*: glutathione S-transferase mu 1; *GPX4*: glutathione peroxidase 4; *AHR*: Aryl hydrocarbon receptor; del: Deletion

* Based on frequency in the ALSPAC population (https://www.ncbi.nlm.nih.gov/snp)

** Formerly rs947894

† High serum level of CC16 (Club cell secretory protein) (14)

†† Missense substitution (I105V): Lower glutathione S-transferase activity leading to genetic susceptibility to oxidative stress; associated with lower lung function growth (15)

§ Deletion (null vs present) –*GSTT1* not expressed: Lower glutathione S-transferase activity leading to genetic susceptibility to oxidative stress; associated with a higher risk of asthma (16)

§§ Deletion (null vs present) –*GSTM1* not expressed: Lower glutathione S-transferase activity leading to genetic susceptibility to oxidative stress; associated with lower lung function growth (15)

‡ Low efficiency in selenocysteine incorporation into GPX4 leading to the altered synthesis of this antioxidant enzyme, particularly when selenium intake is suboptimal (17-19)

‡‡ Lower mRNA expression of *AHR* leading to reduced AHR antioxidant activity and decreased metabolism of dioxins (organic environmental pollutants) by cytochrome P450 (20)

Table S3: Participant* characteristics† according to quartiles of 'health-conscious' dietary pattern score at 7 years of age

	Quartiles of 'health-conscious' dietary pattern				
	**Q1**	**Q2**	**Q3**	**Q4**	**P-value**
n (%)	1267 (23.5)	1362 (25.2)	1386 (25.7)	1385 (25.6)	
Male, n (%)	666 (52.6)	652 (47.9)	673 (48.6)	683 (49.3)	0.08
Index of Multiple Deprivation, n (%)					<0.001
Q1: Least deprived	294 (25.5)	437 (35.2)	457 (36.7)	471 (38.6)	
Q5: Most deprived	169 (14.6)	141 (11.4)	143 (11.5)	154 (12.6)	
Place of residence, n (%)					<0.001
Urban	1009 (79.6)	1023 (75.1)	1018 (73.4)	984 (71.0)	
Rural	153 (12.1)	229 (16.8)	239 (17.2)	247 (17.8)	
Missing	105 (8.3)	110 (8.1)	129 (9.3)	154 (11.1)	
Older siblings, n (%)	640 (50.5)	723 (53.1)	711 (51.3)	717 (51.8)	0.60
Younger siblings, n (%)	663 (52.3)	711 (52.2)	715 (51.6)	717 (51.8)	0.98
Breastfeeding by the 3rd month, n (%)					<0.001
Never	288 (22.7)	227 (16.7)	180 (13.0)	93 (6.7)	
Stopped/Non-exclusive	617 (48.7)	662 (48.6)	640 (46.2)	594 (42.9)	
Exclusive	310 (24.5)	414 (30.4)	504 (36.4)	617 (44.5)	
Missing	52 (4.1)	59 (4.3)	62 (4.5)	81 (5.8)	
Vigorous physical activity, n (%)					<0.001
<3 times a week	585 (46.2)	615 (45.2)	580 (41.8)	510 (36.8)	
4-6 times a week	235 (18.5)	290 (21.3)	316 (22.8)	349 (25.2)	
Daily	106 (8.4)	134 (9.8)	131 (9.5)	168 (12.1)	
Missing	341 (26.9)	323 (23.7)	359 (25.9)	358 (25.8)	
BMI, kg/m^2	16.1 ± 2.0	16.2 ± 2.1	16.2 ± 1.9	16.1 ± 1.9	0.58
BMI at 9 years, kg/m^2	17.6 ± 2.9	17.7 ± 2.9	17.6 ± 2.7	17.4 ± 2.6	0.21
FMI at 9 years, kg/m^x	1.27 ± 0.8	1.30 ± 0.8	1.28 ± 0.8	1.20 ± 0.7	0.01
History of food allergy, n (%)	182 (14.4)	225 (16.5)	241 (17.4)	289 (20.9)	0.001
Atopy, n (%)	175 (19.6)	188 (19.2)	223 (21.1)	231 (21.3)	0.53
Season of dietary information collection, n (%)					0.12
Winter	338 (26.7)	355 (26.1)	368 (26.6)	331 (23.9)	
Spring	397 (31.3)	367 (26.9)	405 (29.2)	411 (29.7)	
Summer	340 (26.8)	399 (29.3)	368 (26.6)	410 (29.6)	
Autumn	187 (14.8)	229 (16.8)	230 (16.6)	210 (15.2)	
Missing	5 (0.4)	12 (0.9)	15 (1.1)	23 (1.7)	0.08
Any supplement use, n (%)	302 (23.8)	459 (33.7)	493 (35.6)	565 (40.8)	<0.001
Total energy intake, kJ/day	6931 ± 1540	7336 ± 1486	7786 ± 1663	8319 ± 1899	<0.001
Total antioxidant capacity, µmol of TE/g/d	6826 ± 1612	7137 ± 1674	7444 ± 1735	8359 ± 2116	<0.001
Vitamin C intake, mg/d	64.1 ± 24.5	72.8 ± 27.7	79.1 ± 31.4	90.3 ± 34.4	<0.001
Vitamin D intake, mg/d	2.78 ± 0.7	2.84 ± 0.7	2.84 ± 0.8	2.92 ± 0.9	<0.001
Vitamin E intake, mg/d	9.62 ± 2.9	9.85 ± 2.8	9.80 ± 2.9	9.98 ± 3.0	0.02
β-carotene intake, µg/d	1790 ± 753	1891 ± 734	1993 ± 852	2199 ± 947	<0.001
Zinc intake, mg/d	6.19 ± 0.9	6.31 ± 0.9	6.45 ± 1.0	6.68 ± 1.0	<0.001

Selenium intake, μg/d	62.6 ± 12.1	63.1 ± 12.8	64.4 ± 13.8	68.6 ± 15.9	<0.001
VLC n-3 PUFA intake from fish, mg/d intake from fish, mg/d	57.9 ± 55.4	73.7 ± 69.6	84.2 ± 85.7	111 ± 115	<0.001
Preformed vitamin A intake, μg/d	442 ± 127	450 ± 118	460 ± 152	469 ± 158	<0.001
dietary fibre intake, g/d	10.2 ± 1.7	11.0 ± 2.0	11.6 ± 2.3	13.2 ± 2.7	<0.001
Maternal smoking, n (%)					0.005
No	974 (76.9)	1097 (80.5)	1123 (81.0)	1130 (81.6)	
Yes	244 (19.3)	209 (15.3)	216 (15.6)	201 (14.5)	
Missing	49 (3.9)	56 (4.1)	47 (3.4)	54 (3.9)	
**Parental factors in pregnancy**					
Maternal age, year	28.6 ± 4.5	29.0 ± 4.2	29.7 ± 4.3	30.2 ± 4.5	<0.001
Maternal education, n (%)					<0.001
Secondary or vocational	379 (29.9)	272 (20.0)	213 (15.4)	147 (10.6)	
O level	522 (41.2)	526 (38.6)	448 (32.3)	354 (25.6)	
A level or degree	346 (27.3)	546 (40.1)	704 (50.8)	869 (62.7)	
Missing	20 (1.6)	18 (1.3)	21 (1.5)	15 (1.1)	
Housing tenure, n (%)					<0.001
Mortgaged/owned	1042 (82.2)	1151 (84.5)	1179 (85.1)	1154 (83.3)	
Council rented	118 (9.3)	83 (6.1)	69 (5.0)	59 (4.3)	
Non-council rented	55 (4.3)	57 (4.2)	74 (5.3)	122 (8.8)	
Missing	52 (4.1)	71 (5.2)	64 (4.6)	50 (3.6)	
Financial difficulty, n (%)					0.13
No	1087 (85.8)	1165 (85.5)	1163 (83.9)	1147 (82.8)	
Yes	175 (13.8)	190 (14.0)	216 (15.6)	229 (16.5)	
Missing	5 (0.4)	7 (0.5)	7 (0.5)	9 (0.6)	
Maternal ethnicity, n (%)					0.006
White	1235 (97.5)	1328 (97.5)	1341 (96.8)	1335 (96.4)	
Non-white	10 (0.8)	16 (1.2)	22 (1.6)	33 (2.4)	
Missing	22 (1.7)	18 (1.3)	23 (1.7)	17 (1.2)	
Maternal history of atopy, n (%)					0.10
No	689 (54.4)	714 (52.4)	700 (50.5)	684 (49.4)	
Yes	537 (42.4)	607 (44.6)	628 (45.3)	644 (46.5)	
Missing	41 (3.2)	41 (3.0)	58 (4.2)	57 (4.1)	
Paternal history of atopy, n (%)					0.34
No	552 (43.6)	587 (43.1)	568 (41.0)	609 (44.0)	
Yes	373 (29.4)	409 (30.0)	449 (32.4)	440 (31.8)	
Missing	342 (27.0)	366 (26.9)	369 (26.6)	336 (24.3)	
Maternal 'Processed' DP score	-0.00 ± 1.0	-0.08 ± 0.9	-0.15 ± 0.9	-0.26 ± 0.9	<0.001
Maternal 'Traditional' DP score	-0.12 ± 0.9	-0.08 ± 0.9	-0.01 ± 0.9	0.17 ± 1.1	<0.001
Maternal 'Healthy' DP score	-0.27 ± 0.8	0.07 ± 0.9	0.36 ± 0.9	0.73 ± 0.9	<0.001

[*] Children included in incident asthma or lung function analysis (n= 5,400).

[†] The characteristics in the table are around 7 years of child age unless otherwise stated, or the item is listed under the 'Parental factors in pregnancy'.

Numbers are mean ± SD unless otherwise specified.

BMI: Body mass index; FMI: Fat mass index; TE: Trolox equivalents; VLC n-3 PUFA: very-long-chain ω-3 polyunsaturated fatty acids; DP: Dietary pattern.

Table S4: Participant[*] characteristics[†] according to quartiles of 'traditional' dietary pattern score at 7 years of age

	Quartiles of 'traditional' dietary pattern				
	**Q1**	**Q2**	**Q3**	**Q4**	**P-value**
n (%)	1378 (25.5)	1355 (25.1)	1324 (24.5)	1343 (24.9)	
Male, n (%)	737 (53.5)	668 (49.3)	650 (49.1)	619 (46.1)	0.002
Index of Multiple Deprivation, n (%)					0.020
Q1: Least deprived	399 (32.2)	414 (33.5)	452 (38.5)	394 (32.5)	
Q5: Most deprived	189 (15.2)	141 (11.4)	123 (10.5)	154 (12.7)	
Place of residence, n (%)					0.001
Urban	1070 (77.6)	1020 (75.3)	936 (70.7)	1008 (75.1)	
Rural	181 (13.1)	226 (16.7)	247 (18.7)	214 (15.9)	
Missing	127 (9.2)	109 (8.0)	141 (10.6)	121 (9.0)	
Older siblings, n (%)	704 (51.1)	705 (52.0)	669 (50.5)	713 (53.1)	0.57
Younger siblings, n (%)	695 (50.4)	679 (50.1)	716 (54.1)	716 (53.3)	0.09
Breastfeeding by the 3rd month, n (%)					0.18
Never	230 (16.7)	195 (14.4)	180 (13.6)	183 (13.6)	
Stopped/Non-exclusive	622 (45.1)	641 (47.3)	610 (46.1)	640 (47.7)	
Exclusive	456 (33.1)	456 (33.7)	475 (35.9)	458 (34.1)	
Missing	70 (5.1)	63 (4.6)	59 (4.5)	62 (4.6)	
Vigorous physical activity, n (%)					0.05
<3 times a week	634 (46.0)	561 (41.4)	571 (43.1)	524 (39.0)	
4-6 times a week	276 (20.0)	290 (21.4)	308 (23.3)	316 (23.5)	
Daily	130 (9.4)	134 (9.9)	133 (10.0)	142 (10.6)	
Missing	338 (24.5)	370 (27.3)	312 (23.6)	361 (26.9)	
BMI, kg/m^2	16.1 ± 2.0	16.1 ± 1.9	16.2 ± 1.9	16.2 ± 1.9	0.41
BMI at 9 years, kg/m^2	17.6 ± 2.9	17.5 ± 2.7	17.6 ± 2.8	17.7 ± 2.7	0.39
FMI at 9 years, kg/m^x	1.25 ± 0.8	1.25 ± 0.8	1.25 ± 0.8	1.30 ± 0.8	0.33
History of food allergy, n (%)	233 (16.9)	230 (17.0)	244 (18.4)	230 (17.1)	0.61
Atopy, n (%)	209 (20.5)	208 (21.2)	211 (21.3)	189 (18.7)	0.44
Season of dietary information collection, n (%)					0.39
Winter	333 (24.2)	345 (25.5)	361 (27.3)	353 (26.3)	
Spring	419 (30.4)	410 (30.3)	368 (27.8)	383 (28.5)	
Summer	407 (29.5)	385 (28.4)	357 (27.0)	368 (27.4)	
Autumn	202 (14.7)	203 (15.0)	225 (17.0)	226 (16.8)	
Missing	17 (1.2)	12 (0.9)	13 (1.0)	13 (1.0)	
Any supplement use, n (%)	521 (37.8)	458 (33.8)	434 (32.8)	406 (30.2)	<0.001
Total energy intake, kJ/day	6980 ± 1642	7342 ± 1529	7669 ± 1560	8457 ± 1838	<0.001
Total antioxidant capacity, µmol of TE/g/d	7173 ± 1982	7231 ± 1749	7415 ± 1670	8003 ± 2002	<0.001
Vitamin C intake, mg/d	69.2 ± 30.3	72.7 ± 29.9	77.4 ± 28.7	88.3 ± 32.8	<0.001
Vitamin D intake, mg/d	2.70 ± 0.8	2.81 ± 0.7	2.86 ± 0.7	3.03 ± 0.9	<0.001
Vitamin E intake, mg/d	9.98 ± 3.2	9.79 ± 2.8	9.78 ± 2.8	9.71 ± 2.9	0.10
β-carotene intake, µg/d	1516 ± 728	1840 ± 671	2041 ± 700	2508 ± 918	<0.001
Zinc intake, mg/d	5.83 ± 0.9	6.33 ± 0.8	6.50 ± 0.8	7.00 ± 1.0	<0.001

Selenium intake, μg/d	62.1 ± 14.0	63.5 ± 13.1	65.2 ± 12.7	68.2 ± 15.1	<0.001
VLC n-3 PUFA intake from fish, mg/d	57.0 ± 61.9	72.9 ± 70.4	86.3 ± 81.9	113 ± 115	<0.001
Preformed vitamin A intake, μg/d	448 ± 135	445 ± 125	460 ± 140	469 ± 158	<0.001
dietary fibre intake, g/d	10.7 ± 2.6	11.3 ± 2.3	11.7 ± 2.1	12.4 ± 2.5	<0.001
Maternal smoking, n (%)					0.20
No	1098 (79.7)	1099 (81.1)	1078 (81.4)	1049 (78.1)	
Yes	235 (17.1)	208 (15.4)	196 (14.8)	231 (17.2)	
Missing	45 (3.3)	48 (3.5)	50 (3.8)	63 (4.7)	
**Parental factors in pregnancy**					
Maternal age, year	29.6 ± 4.5	29.5 ± 4.4	29.4 ± 4.3	29.0 ± 4.5	0.002
Maternal education, n (%)					0.13
Secondary or vocational	288 (20.9)	246 (18.2)	224 (16.9)	253 (18.8)	
O level	459 (33.3)	451 (33.3)	462 (34.9)	478 (35.6)	
A level or degree	613 (44.5)	640 (47.2)	619 (46.8)	593 (44.2)	
Missing	18 (1.3)	18 (1.3)	19 (1.4)	19 (1.4)	
Housing tenure, n (%)					<0.001
Mortgaged/owned	1115 (80.9)	1157 (85.4)	1121 (84.7)	1133 (84.4)	
Council rented	93 (6.7)	79 (5.8)	78 (5.9)	79 (5.9)	
Non-council rented	114 (8.3)	65 (4.8)	62 (4.7)	67 (5.0)	
Missing	56 (4.1)	54 (4.0)	63 (4.8)	64 (4.8)	
Financial difficulty, n (%)					0.04
No	1154 (83.7)	1160 (85.6)	1138 (86.0)	1110 (82.7)	
Yes	215 (15.6)	187 (13.8)	179 (13.5)	229 (17.1)	
Missing	9 (0.7)	8 (0.6)	7 (0.5)	<5	
Maternal ethnicity, n (%)					0.32
White	1337 (97.0)	1318 (97.3)	1285 (97.1)	1299 (96.7)	
Non-white	26 (1.9)	17 (1.3)	15 (1.1)	23 (1.7)	
Missing	15 (1.1)	20 (1.5)	24 (1.8)	21 (1.6)	
Maternal history of atopy, n (%)					0.43
No	716 (52.0)	701 (51.7)	660 (49.8)	710 (52.9)	
Yes	619 (44.9)	603 (44.5)	614 (46.4)	580 (43.2)	
Missing	43 (3.1)	51 (3.8)	50 (3.8)	53 (3.9)	
Paternal history of atopy, n (%)					0.29
No	565 (41.0)	593 (43.8)	580 (43.8)	578 (43.0)	
Yes	452 (32.8)	418 (30.8)	407 (30.7)	394 (29.3)	
Missing	361 (26.2)	344 (25.4)	337 (25.5)	371 (27.6)	
Maternal ‘Processed’ DP score	-0.15 ± 1.0	-0.15 ± 0.9	-0.12 ± 0.8	-0.08 ± 0.9	0.12
Maternal ‘Traditional’ DP score	-0.24 ± 0.9	-0.13 ± 0.9	0.02 ± 0.9	0.35 ± 1.1	<0.001
Maternal ‘Healthy’ DP score	0.15 ± 1.0	0.21 ± 0.9	0.26 ± 0.9	0.31 ± 1.0	<0.001

[*] Children included in incident asthma or lung function analysis (n= 5,400).

[†] The characteristics in the table are at around 7 years of child age unless otherwise stated, or the item is listed under the ‘Parental factors in pregnancy’.

Numbers are mean ± SD unless otherwise specified.

BMI: Body mass index; FMI: Fat mass index; TE: Trolox equivalents; VLC n-3 PUFA: very-long-chain ω-3 polyunsaturated fatty acids; DP: Dietary pattern.

Table S5: Participant* characteristics† according to quartiles of 'processed' dietary pattern score at 7 years of age

	Quartiles of 'processed' dietary pattern				
	**Q1**	**Q2**	**Q3**	**Q4**	**P-value**
n (%)	1449 (26.8)	1394 (25.8)	1335 (24.7)	1222 (22.6)	
Male, n (%)	678 (46.8)	690 (49.5)	672 (50.3)	634 (51.9)	0.060
Index of Multiple Deprivation, n (%)					<0.001
Q1: Least deprived	520 (40.5)	449 (36.0)	400 (33.0)	290 (26.0)	
Q5: Most deprived	144 (11.2)	149 (11.9)	145 (12.0)	169 (15.1)	
Place of residence, n (%)					<0.001
Urban	1006 (69.4)	1031 (74.0)	1018 (76.3)	979 (80.1)	
Rural	295 (20.4)	224 (16.1)	205 (15.4)	144 (11.8)	
Missing	148 (10.2)	139 (10.0)	112 (8.4)	99 (8.1)	
Older siblings, n (%)	718 (49.6)	714 (51.2)	713 (53.4)	646 (52.9)	0.170
Younger siblings, n (%)	749 (51.7)	699 (50.1)	699 (52.4)	659 (53.9)	0.276
Breastfeeding by the 3rd month, n (%)					<0.001
Never	131 (9.0)	168 (12.1)	229 (17.2)	260 (21.3)	
Stopped/Non-exclusive	630 (43.5)	651 (46.7)	632 (47.3)	600 (49.1)	
Exclusive	620 (42.8)	523 (37.5)	403 (30.2)	299 (24.5)	
Missing	68 (4.7)	52 (3.7)	71 (5.3)	63 (5.2)	
Vigorous physical activity, n (%)					0.117
<3 times a week	590 (40.7)	604 (43.3)	570 (42.7)	526 (43.0)	
4-6 times a week	361 (24.9)	314 (22.5)	272 (20.4)	243 (19.9)	
Daily	146 (10.1)	137 (9.8)	138 (10.3)	118 (9.7)	
Missing	352 (24.3)	339 (24.3)	355 (26.6)	335 (27.4)	
BMI, kg/m^2	16.1 ± 1.9	16.1 ± 1.9	16.2 ± 2.0	16.3 ± 2.1	0.134
BMI at 9 years, kg/m^2	17.4 ± 2.6	17.6 ± 2.7	17.6 ± 2.8	17.7 ± 2.9	0.019
FMI at 9 years, kg/m^x	1.23 ± 0.7	1.27 ± 0.8	1.28 ± 0.8	1.28 ± 0.8	0.302
History of food allergy, n (%)	324 (22.4)	234 (16.8)	196 (14.7)	183 (15.0)	<0.001
Atopy, n (%)	224 (20.6)	217 (20.6)	196 (19.6)	180 (20.6)	0.930
Season of dietary information collection, n (%)					<0.001
Winter	411 (28.4)	354 (25.4)	342 (25.6)	285 (23.3)	
Spring	424 (29.3)	440 (31.6)	369 (27.6)	347 (28.4)	
Summer	354 (24.4)	361 (25.9)	390 (29.2)	412 (33.7)	
Autumn	243 (16.8)	225 (16.1)	222 (16.6)	166 (13.6)	
Missing	17 (1.2)	14 (1.0)	12 (0.9)	12 (1.0)	
Any supplement use, n (%)	539 (37.2)	476 (34.1)	433 (32.4)	371 (30.4)	0.002
Total energy intake, kJ/day	6592 ± 1417	7191 ± 1377	7821 ± 1379	9046 ± 1773	<0.001
Total antioxidant capacity, µmol of TE/g/d	7894 ± 1937	7340 ± 1742	7238 ± 1792	7297 ± 1997	<0.001
Vitamin C intake, mg/d	83.8 ± 31.1	77.8 ± 29.9	73.7 ± 30.2	70.7 ± 32.5	<0.001
Vitamin D intake, mg/d	2.93 ± 0.8	2.91 ± 0.8	2.82 ± 0.8	2.71 ± 0.8	<0.001
Vitamin E intake, mg/d	9.91 ± 2.9	9.88 ± 2.9	9.81 ± 2.8	9.65 ± 3.0	0.11
β-carotene intake, µg/d	2326 ± 852	2039 ± 790	1825 ± 745	1635 ± 811	<0.001
Zinc intake, mg/d	6.81 ± 0.9	6.48 ± 0.9	6.27 ± 0.9	6.00 ± 1.0	<0.001

Selenium intake, μg/d	68.7 ± 14.2	66.1 ± 13.3	63.4 ± 12.7	59.8 ± 14.0	<0.001
VLC n-3 PUFA intake from fish, mg/d	98.0 ± 96.9	83.1 ± 78.3	78.8 ± 86.2	65.5 ± 81.2	<0.001
Preformed vitamin A intake, μg/d	485 ± 125	462 ± 128	440 ± 118	429 ± 182	<0.001
dietary fibre intake, g/d	12.9 ± 2.5	11.6 ± 2.2	11.1 ± 2.1	10.3 ± 2.2	<0.001
Maternal smoking, n (%)					<0.001
No	1203 (83.0)	1162 (83.4)	1041 (78.0)	918 (75.1)	
Yes	193 (13.3)	193 (13.8)	236 (17.7)	248 (20.3)	
Missing	53 (3.7)	39 (2.8)	58 (4.3)	56 (4.6)	
**Parental factors in pregnancy**					
Maternal age, year	30.2 ± 4.4	29.8 ± 4.3	29.1 ± 4.4	28.3 ± 4.4	<0.001
Maternal education, n (%)					<0.001
Secondary or vocational	199 (13.7)	231 (16.6)	259 (19.4)	322 (26.4)	
O level	374 (25.8)	451 (32.4)	527 (39.5)	498 (40.8)	
A level or degree	864 (59.6)	691 (49.6)	529 (39.6)	381 (31.2)	
Missing	12 (0.8)	21 (1.5)	20 (1.5)	21 (1.7)	
Housing tenure, n (%)					<0.001
Mortgaged/owned	1220 (84.2)	1192 (85.5)	1127 (84.4)	987 (80.8)	
Council rented	65 (4.5)	73 (5.2)	78 (5.8)	113 (9.2)	
Non-council rented	103 (7.1)	76 (5.5)	64 (4.8)	65 (5.3)	
Missing	61 (4.2)	53 (3.8)	66 (4.9)	57 (4.7)	
Financial difficulty, n (%)					0.002
No	1226 (84.6)	1214 (87.1)	1122 (84.0)	1000 (81.8)	
Yes	216 (14.9)	173 (12.4)	205 (15.4)	216 (17.7)	
Missing	7 (0.5)	7 (0.5)	8 (0.6)	6 (0.5)	
Maternal ethnicity, n (%)					0.03
White	1399 (96.5)	1352 (97.0)	1299 (97.3)	1189 (97.3)	
Non-white	33 (2.3)	21 (1.5)	14 (1.0)	13 (1.1)	
Missing	17 (1.2)	21 (1.5)	22 (1.6)	20 (1.6)	
Maternal history of atopy, n (%)					0.11
No	713 (49.2)	723 (51.9)	704 (52.7)	647 (52.9)	
Yes	688 (47.5)	622 (44.6)	571 (42.8)	535 (43.8)	
Missing	48 (3.3)	49 (3.5)	60 (4.5)	40 (3.3)	
Paternal history of atopy, n (%)					0.02
No	644 (44.4)	562 (40.3)	570 (42.7)	540 (44.2)	
Yes	468 (32.3)	470 (33.7)	389 (29.1)	344 (28.2)	
Missing	337 (23.3)	362 (26.0)	376 (28.2)	338 (27.7)	
Maternal 'Processed' DP score	-0.42 ± 0.7	-0.19 ± 0.9	-0.04 ± 0.8	0.21 ± 1.0	<0.001
Maternal 'Traditional' DP score	0.05 ± 1.0	-0.02 ± 0.9	-0.03 ± 0.9	-0.03 ± 1.0	0.06
Maternal 'Healthy' DP score	0.46 ± 0.9	0.29 ± 1.0	0.12 ± 0.9	0.01 ± 0.9	<0.001

[*] Children included in incident asthma or lung function analysis (n= 5,400).

[†] The characteristics in the table are at around 7 years of child age unless otherwise stated, or the item is listed under the 'Parental factors in pregnancy'.

Numbers are mean ± SD unless otherwise specified.

BMI: Body mass index; FMI: Fat mass index; TE: Trolox equivalents; VLC n-3 PUFA: very-long-chain ω-3 polyunsaturated fatty acids; DP: Dietary pattern.

Table S6: Linear regression coefficients (95% confidence interval) for pre-bronchodilator lung function measures (z scores) at 15.5 years according to quartiles of dietary pattern scores at 7 years, adjusted for potential confounders

	Quartiles of dietary pattern score				P for trend*	Per SD
	**Q1**	**Q2**	**Q3**	**Q4**		
**'Health-conscious' dietary pattern**						
**FEV_1**						
Model 1	0.00	0.05 (-0.07, 0.17)	0.09 (-0.03, 0.22)	0.12 (-0.00, 0.25)	0.05	0.03 (-0.01, 0.07)
Model 2	0.00	0.05 (-0.07, 0.17)	0.10 (-0.03, 0.23)	0.13 (-0.00, 0.27)	0.05	0.03 (-0.02, 0.08)
**FVC**						
Model 1	0.00	0.04 (-0.08, 0.16)	0.07 (-0.05, 0.19)	0.14 (0.02, 0.26)	0.02	0.04 (-0.00, 0.09)
Model 2	0.00	0.05 (-0.07, 0.17)	0.09 (-0.04, 0.21)	0.16 (0.02, 0.29)	0.02	0.05 (-0.00, 0.09)
**FEV_1/FVC ratio**						
Model 1	0.00	0.04 (-0.07, 0.16)	0.02 (-0.10, 0.13)	0.02 (-0.10, 0.13)	0.98	-0.01 (-0.05, 0.03)
Model 2	0.00	0.04 (-0.08, 0.15)	0.02 (-0.10, 0.14)	0.01 (-0.11, 0.14)	0.97	-0.01 (-0.06, 0.03)
**$FEF_{25–75}$**						
Model 1	0.00	0.06 (-0.05, 0.17)	0.08 (-0.03, 0.19)	0.11 (-0.00, 0.22)	0.07	0.03 (-0.01, 0.06)
Model 2	0.00	0.06 (-0.05, 0.17)	0.09 (-0.03, 0.20)	0.11 (-0.01, 0.23)	0.10	0.02 (-0.02, 0.06)
**'Traditional' dietary pattern**						
**FEV_1**						
Model 1	0.00	0.06 (-0.06, 0.18)	0.07 (-0.05, 0.20)	0.05 (-0.07, 0.18)	0.40	0.02 (-0.02, 0.07)
Model 2	0.00	0.06 (-0.06, 0.18)	0.07 (-0.05, 0.20)	0.05 (-0.08, 0.18)	0.42	0.02 (-0.02, 0.07)
**FVC**						
Model 1	0.00	0.01 (-0.11, 0.13)	0.08 (-0.04, 0.19)	0.06 (-0.06, 0.18)	0.24	0.04 (-0.01, 0.08)
Model 2	0.00	0.01 (-0.11, 0.13)	0.07 (-0.05, 0.19)	0.06 (-0.07, 0.18)	0.26	0.04 (-0.01, 0.08)
**FEV_1/FVC ratio**						
Model 1	0.00	0.05 (-0.06, 0.17)	-0.01 (-0.12, 0.11)	-0.05 (-0.17, 0.07)	0.32	-0.03 (-0.08, 0.01)
Model 2	0.00	0.05 (-0.07, 0.16)	-0.01 (-0.12, 0.11)	-0.05 (-0.17, 0.07)	0.32	-0.03 (-0.08, 0.01)
**$FEF_{25–75}$**						
Model 1	0.00	0.06 (-0.05, 0.17)	0.01 (-0.10, 0.12)	-0.01 (-0.12, 0.10)	0.67	-0.01 (-0.05, 0.03)
Model 2	0.00	0.05 (-0.05, 0.16)	0.01 (-0.10, 0.12)	-0.01 (-0.13, 0.10)	0.66	-0.01 (-0.05, 0.03)
**'Processed' dietary pattern**						
**FEV_1**						
Model 1	0.00	0.04 (-0.08, 0.15)	-0.07 (-0.19, 0.05)	-0.08 (-0.22, 0.06)	0.14	-0.04 (-0.09, 0.02)
Model 2	0.00	0.03 (-0.09, 0.15)	-0.07 (-0.19, 0.06)	-0.08 (-0.23, 0.07)	0.17	-0.03 (-0.09, 0.02)
**FVC**						
Model 1	0.00	-0.02 (-0.13, 0.10)	-0.09 (-0.21, 0.02)	-0.12 (-0.26, 0.01)	0.05	-0.04 (-0.10, 0.01)
Model 5	0.00	-0.02 (-0.14, 0.09)	-0.10 (-0.23, 0.02)	-0.13 (-0.28, 0.01)	0.05	-0.04 (-0.10, 0.01)
**FEV_1/FVC ratio**						
Model 1	0.00	0.10 (-0.01, 0.21)	0.08 (-0.03, 0.20)	0.11 (-0.02, 0.24)	0.13	0.03 (-0.03, 0.08)
Model 2	0.00	0.10 (-0.01, 0.21)	0.09 (-0.03, 0.21)	0.12 (-0.02, 0.26)	0.10	0.04 (-0.02, 0.09)
**$FEF_{25–75}$**						
Model 1	0.00	0.07 (-0.03, 0.17)	-0.00 (-0.11, 0.10)	0.01 (-0.12, 0.13)	0.84	-0.01 (-0.06, 0.04)
Model 2	0.00	0.07 (-0.03, 0.17)	0.00 (-0.11, 0.12)	0.02 (-0.11, 0.15)	0.97	-0.00 (-0.06, 0.05)

* Linear trend was tested by treating the median values of quartiles as a continuous variable
FEV_1: forced expiratory volume in 1s; FVC: forced vital capacity; $FEF_{25–75}$: forced expiratory flow at 25–75% of FVC
Multivariable model 1: sex and total energy intake;
Multivariable model 2: further adjusted for maternal education, housing tenure at birth, financial difficulty during pregnancy, maternal ethnicity, index of multiple deprivation, rural living location, maternal and paternal history of atopic disease, older sibling, younger sibling, breastfeeding, maternal smoking, vigorous physical activity, history of food allergy, and season when the FFQ was completed.

Table S7: Linear regression coefficients (95% confidence interval) for post-bronchodilator lung function measures (z scores) at 15.5 years according to quartiles of dietary pattern scores at 7 years, adjusted for additional potential confounders as well as exclusions (sensitivity analyses)

	Quartiles of dietary pattern score				P for	Per SD
	**Q1**	**Q2**	**Q3**	**Q4**	**trend***	
**'Health-conscious' dietary pattern**						
**FEV_1**						
Model 3	0.00	0.08 (-0.06, 0.22)	0.09 (-0.05, 0.24)	0.17 (0.01, 0.32)	0.04	0.05 (-0.01, 0.10)
Model 4	0.00	0.07 (-0.07, 0.22)	0.09 (-0.06, 0.24)	0.18 (0.01, 0.34)	0.04	0.06 (-0.00, 0.12)
Model 5	0.00	0.08 (-0.06, 0.22)	0.09 (-0.05, 0.24)	0.17 (0.01, 0.32)	0.04	0.05 (-0.01, 0.10)
Model 6	0.00	0.09 (-0.05, 0.23)	0.09 (-0.05, 0.24)	0.17 (0.01, 0.32)	0.04	0.04 (-0.01, 0.10)
Model 7	0.00	0.10 (-0.05, 0.25)	0.10 (-0.05, 0.25)	0.15 (-0.01, 0.31)	0.09	0.04 (-0.01, 0.10)
**FVC**						
Model 3	0.00	0.08 (-0.06, 0.21)	0.07 (-0.07, 0.21)	0.18 (0.03, 0.33)	0.03	0.06 (0.01, 0.11)
Model 4	0.00	0.07 (-0.07, 0.21)	0.07 (-0.07, 0.22)	0.18 (0.02, 0.33)	0.03	0.07 (0.01, 0.12)
Model 5	0.00	0.08 (-0.05, 0.22)	0.08 (-0.06, 0.22)	0.19 (0.04, 0.33)	0.02	0.06 (0.01, 0.12)
Model 6	0.00	0.08 (-0.06, 0.22)	0.08 (-0.06, 0.22)	0.18 (0.03, 0.33)	0.02	0.06 (0.01, 0.11)
Model 7	0.00	0.10 (-0.04, 0.24)	0.08 (-0.06, 0.22)	0.18 (0.03, 0.33)	0.03	0.06 (0.01, 0.12)
**FEV_1/FVC ratio**						
Model 3	0.00	-0.01 (-0.13, 0.11)	0.01 (-0.11, 0.13)	-0.05 (-0.18, 0.09)	0.52	-0.04 (-0.08, 0.01)
Model 4	0.00	-0.01 (-0.13, 0.11)	0.00 (-0.12, 0.13)	-0.04 (-0.18, 0.10)	0.57	-0.03 (-0.08, 0.02)
Model 5	0.00	-0.02 (-0.14, 0.10)	0.01 (-0.12, 0.13)	-0.06 (-0.19, 0.07)	0.40	-0.04 (-0.09, 0.01)
Model 6	0.00	-0.00 (-0.12, 0.12)	-0.00 (-0.13, 0.12)	-0.05 (-0.18, 0.08)	0.43	-0.04 (-0.09, 0.01)
Model 7	0.00	-0.01 (-0.14, 0.11)	0.02 (-0.11, 0.15)	-0.09 (-0.23, 0.05)	0.20	-0.04 (-0.09, 0.01)
**$FEF_{25–75}$**						
Model 3	0.00	0.07 (-0.05, 0.20)	0.05 (-0.07, 0.18)	0.10 (-0.04, 0.23)	0.22	0.03 (-0.02, 0.07)
Model 4	0.00	0.08 (-0.05, 0.20)	0.05 (-0.08, 0.17)	0.10 (-0.04, 0.24)	0.21	0.03 (-0.02, 0.08)
Model 5	0.00	0.08 (-0.04, 0.20)	0.05 (-0.07, 0.18)	0.10 (-0.03, 0.23)	0.22	0.03 (-0.02, 0.07)
Model 6	0.00	0.09 (-0.03, 0.21)	0.05 (-0.07, 0.18)	0.10 (-0.03, 0.23)	0.23	0.02 (-0.02, 0.07)
Model 7	0.00	0.08 (-0.05, 0.20)	0.06 (-0.07, 0.19)	0.07 (-0.07, 0.21)	0.45	0.02 (-0.03, 0.07)
**'Traditional' dietary pattern**						
**FEV_1**						
Model 3	0.00	0.07 (-0.07, 0.21)	0.12 (-0.02, 0.27)	0.01 (-0.13, 0.16)	0.70	0.01 (-0.05, 0.07)
Model 4	0.00	0.07 (-0.07, 0.20)	0.15 (0.01, 0.29)	0.05 (-0.09, 0.20)	0.32	0.02 (-0.03, 0.08)
Model 5	0.00	0.06 (-0.08, 0.20)	0.12 (-0.01, 0.26)	0.02 (-0.12, 0.16)	0.59	0.01 (-0.04, 0.07)
Model 6	0.00	0.07 (-0.07, 0.21)	0.11 (-0.03, 0.25)	0.01 (-0.14, 0.15)	0.80	0.00 (-0.05, 0.06)
Model 7	0.00	0.06 (-0.09, 0.20)	0.14 (0.00, 0.29)	0.05 (-0.10, 0.20)	0.37	0.03 (-0.03, 0.09)
**FVC**						
Model 3	0.00	0.05 (-0.08, 0.18)	0.08 (-0.05, 0.22)	0.00 (-0.14, 0.14)	0.87	0.01 (-0.05, 0.06)
Model 4	0.00	0.05 (-0.08, 0.18)	0.11 (-0.02, 0.24)	0.05 (-0.09, 0.19)	0.36	0.03 (-0.03, 0.08)
Model 5	0.00	0.04 (-0.09, 0.17)	0.09 (-0.04, 0.22)	0.02 (-0.12, 0.16)	0.65	0.02 (-0.04, 0.07)
Model 6	0.00	0.05 (-0.08, 0.18)	0.07 (-0.06, 0.21)	0.02 (-0.12, 0.16)	0.73	0.01 (-0.04, 0.06)
Model 7	0.00	0.04 (-0.10, 0.18)	0.11 (-0.03, 0.25)	0.05 (-0.10, 0.19)	0.38	0.03 (-0.02, 0.09)
**FEV_1/FVC ratio**						
Model 3	0.00	-0.02 (-0.14, 0.10)	-0.01 (-0.13, 0.11)	-0.02 (-0.15, 0.10)	0.76	-0.01 (-0.06, 0.04)
Model 4	0.00	-0.02 (-0.14, 0.10)	-0.01 (-0.13, 0.11)	-0.05 (-0.17, 0.08)	0.51	-0.02 (-0.06, 0.03)

Model 5	0.00	-0.02 (-0.14, 0.09)	-0.02 (-0.13, 0.10)	-0.04 (-0.16, 0.08)	0.53	-0.02 (-0.06, 0.03)
Model 6	0.00	-0.01 (-0.13, 0.11)	-0.01 (-0.13, 0.11)	-0.05 (-0.17, 0.08)	0.48	-0.02 (-0.07, 0.02)
Model 7	0.00	-0.03 (-0.15, 0.09)	-0.03 (-0.15, 0.10)	-0.05 (-0.18, 0.08)	0.48	-0.02 (-0.06, 0.03)
**$FEF_{25–75}$**						
Model 3	0.00	0.04 (-0.08, 0.15)	0.04 (-0.09, 0.16)	-0.04 (-0.17, 0.09)	0.57	-0.02 (-0.07, 0.03)
Model 4	0.00	0.03 (-0.08, 0.15)	0.05 (-0.07, 0.17)	-0.03 (-0.15, 0.10)	0.75	-0.01 (-0.06, 0.04)
Model 5	0.00	0.03 (-0.08, 0.15)	0.03 (-0.08, 0.15)	-0.04 (-0.16, 0.09)	0.59	-0.02 (-0.06, 0.03)
Model 6	0.00	0.04 (-0.08, 0.16)	0.03 (-0.09, 0.15)	-0.05 (-0.17, 0.08)	0.44	-0.02 (-0.07, 0.02)
Model 7	0.00	0.03 (-0.09, 0.16)	0.04 (-0.08, 0.17)	-0.03 (-0.16, 0.10)	0.71	-0.01 (-0.05, 0.04)
**'Processed' dietary pattern**						
**FEV_1**						
Model 3	0.00	0.06 (-0.07, 0.20)	-0.01 (-0.16, 0.14)	-0.09 (-0.26, 0.09)	0.26	-0.02 (-0.09, 0.05)
Model 4	0.00	0.05 (-0.08, 0.18)	-0.05 (-0.19, 0.09)	-0.12 (-0.29, 0.05)	0.11	-0.04 (-0.11, 0.03)
Model 5	0.00	0.04 (-0.09, 0.18)	-0.04 (-0.18, 0.10)	-0.12 (-0.29, 0.04)	0.11	-0.04 (-0.11, 0.03)
Model 6	0.00	0.06 (-0.08, 0.19)	-0.05 (-0.20, 0.09)	-0.13 (-0.29, 0.04)	0.09	-0.04 (-0.11, 0.03)
Model 7	0.00	0.06 (-0.07, 0.20)	-0.04 (-0.19, 0.11)	-0.12 (-0.29, 0.05)	0.12	-0.04 (-0.11, 0.03)
**FVC**						
Model 3	0.00	0.03 (-0.09, 0.16)	-0.04 (-0.18, 0.10)	-0.14 (-0.30, 0.03)	0.09	-0.04 (-0.10, 0.03)
Model 4	0.00	0.03 (-0.10, 0.15)	-0.07 (-0.21, 0.07)	-0.16 (-0.32, 0.01)	0.04	-0.05 (-0.11, 0.02)
Model 5	0.00	0.02 (-0.11, 0.15)	-0.06 (-0.20, 0.07)	-0.17 (-0.33, -0.01)	0.03	-0.05 (-0.12, 0.01)
Model 6	0.00	0.03 (-0.10, 0.16)	-0.07 (-0.20, 0.07)	-0.15 (-0.31, 0.01)	0.04	-0.04 (-0.11, 0.02)
Model 7	0.00	0.04 (-0.09, 0.18)	-0.05 (-0.19, 0.09)	-0.16 (-0.32, 0.01)	0.04	-0.05 (-0.12, 0.02)
**FEV_1/FVC ratio**						
Model 3	0.00	0.01 (-0.11, 0.12)	0.05 (-0.08, 0.17)	0.10 (-0.05, 0.25)	0.18	0.03 (-0.03, 0.10)
Model 4	0.00	0.01 (-0.10, 0.12)	0.05 (-0.07, 0.17)	0.10 (-0.04, 0.24)	0.16	0.04 (-0.02, 0.09)
Model 5	0.00	0.01 (-0.10, 0.12)	0.06 (-0.06, 0.18)	0.11 (-0.03, 0.25)	0.11	0.04 (-0.02, 0.10)
Model 6	0.00	0.02 (-0.09, 0.13)	0.04 (-0.08, 0.17)	0.10 (-0.04, 0.24)	0.16	0.04 (-0.02, 0.09)
Model 7	0.00	-0.00 (-0.12, 0.11)	0.04 (-0.09, 0.16)	0.10 (-0.05, 0.24)	0.17	0.03 (-0.03, 0.09)
**$FEF_{25–75}$**						
Model 3	0.00	0.02 (-0.10, 0.13)	-0.01 (-0.13, 0.12)	-0.03 (-0.18, 0.12)	0.62	-0.02 (-0.08, 0.04)
Model 4	0.00	0.02 (-0.09, 0.13)	-0.02 (-0.14, 0.10)	-0.04 (-0.19, 0.10)	0.50	-0.02 (-0.08, 0.03)
Model 5	0.00	0.02 (-0.10, 0.13)	-0.01 (-0.13, 0.11)	-0.04 (-0.18, 0.11)	0.58	-0.02 (-0.08, 0.04)
Model 6	0.00	0.02 (-0.09, 0.14)	-0.02 (-0.15, 0.10)	-0.05 (-0.19, 0.10)	0.45	-0.02 (-0.08, 0.03)
Model 7	0.00	0.01 (-0.10, 0.13)	-0.03 (-0.16, 0.10)	-0.03 (-0.18, 0.11)	0.55	-0.03 (-0.09, 0.04)

[*] Linear trend was tested by treating the median values of quartiles as a continuous variable

FEV_1: forced expiratory volume in 1s; FVC: forced vital capacity; $FEF_{25–75}$: forced expiratory flow at 25–75% of FVC

Benchmark model (multivariable model 2): sex, total energy intake, maternal education, housing tenure at birth, financial difficulty during pregnancy, maternal ethnicity, index of multiple deprivation, rural living location, maternal and paternal history of atopic disease, older sibling, younger sibling, breastfeeding, maternal smoking, vigorous physical activity, history of food allergy, and season when the FFQ was completed;

Multivariable model 3: further adjusted for the other two dietary patterns (mutual adjustment);

Multivariable model 4: further adjusted for the corresponding maternal dietary pattern in pregnancy;

Multivariable model 5: further adjusted for any supplement use;

Multivariable model 6: model 2 excluding children of non-white mothers;

Multivariable model 7: model 2 excluding children with extreme energy intakes.

Table S8: Linear regression coefficients (95% confidence interval) for pre-bronchodilator lung function measures (z scores) at 8 years according to quartiles of dietary pattern scores at 7 years, adjusted for potential confounders

	Quartiles of dietary pattern score				P for trend*	Per SD
	Q1	Q2	Q3	Q4		
**'Health-conscious' dietary pattern**						
**FEV_1**						
Model 1	0.00	0.07 (-0.04, 0.18)	0.12 (0.00, 0.23)	0.18 (0.06, 0.29)	0.002	0.07 (0.03, 0.11)
Model 2	0.00	0.03 (-0.08, 0.15)	0.07 (-0.05, 0.19)	0.10 (-0.03, 0.22)	0.11	0.04 (-0.01, 0.08)
**FVC**						
Model 1	0.00	0.08 (-0.03, 0.20)	0.16 (0.05, 0.27)	0.22 (0.10, 0.33)	<0.001	0.08 (0.04, 0.12)
Model 2	0.00	0.06 (-0.05, 0.18)	0.13 (0.02, 0.25)	0.18 (0.05, 0.30)	0.004	0.07 (0.03, 0.11)
**FEV_1/FVC ratio**						
Model 1	0.00	-0.05 (-0.16, 0.07)	-0.06 (-0.18, 0.05)	-0.05 (-0.18, 0.07)	0.43	-0.03 (-0.07, 0.01)
Model 2	0.00	-0.08 (-0.20, 0.04)	-0.11 (-0.23, 0.02)	-0.13 (-0.26, -0.00)	0.06	-0.05 (-0.10, -0.01)
**$FEF_{25–75}$**						
Model 1	0.00	0.08 (-0.03, 0.20)	0.08 (-0.04, 0.19)	0.10 (-0.01, 0.22)	0.12	0.03 (-0.01, 0.07)
Model 2	0.00	0.05 (-0.06, 0.17)	0.04 (-0.08, 0.16)	0.03 (-0.10, 0.15)	0.84	-0.00 (-0.04, 0.04)
**'Traditional' dietary pattern**						
**FEV_1**						
Model 1	0.00	0.07 (-0.04, 0.18)	0.01 (-0.10, 0.12)	0.05 (-0.06, 0.17)	0.52	0.01 (-0.03, 0.05)
Model 2	0.00	0.06 (-0.05, 0.17)	0.01 (-0.10, 0.12)	0.05 (-0.07, 0.16)	0.58	0.01 (-0.03, 0.05)
**FVC**						
Model 1	0.00	0.07 (-0.04, 0.18)	0.07 (-0.04, 0.18)	0.06 (-0.06, 0.17)	0.34	0.01 (-0.03, 0.05)
Model 2	0.00	0.05 (-0.06, 0.16)	0.06 (-0.05, 0.17)	0.04 (-0.07, 0.16)	0.46	0.01 (-0.03, 0.05)
**FEV_1/FVC ratio**						
Model 1	0.00	-0.00 (-0.12, 0.11)	-0.09 (-0.21, 0.02)	-0.03 (-0.15, 0.09)	0.41	-0.01 (-0.05, 0.03)
Model 2	0.00	0.01 (-0.11, 0.13)	-0.09 (-0.20, 0.03)	-0.02 (-0.14, 0.10)	0.50	-0.00 (-0.05, 0.04)
**$FEF_{25–75}$**						
Model 1	0.00	0.07 (-0.04, 0.18)	-0.01 (-0.12, 0.10)	0.04 (-0.08, 0.16)	0.76	0.01 (-0.04, 0.05)
Model 2	0.00	0.07 (-0.04, 0.18)	-0.01 (-0.12, 0.10)	0.04 (-0.07, 0.16)	0.69	0.01 (-0.03, 0.06)
**'Processed' dietary pattern**						
**FEV_1**						
Model 1	0.00	-0.01 (-0.11, 0.10)	0.00 (-0.11, 0.12)	-0.07 (-0.20, 0.06)	0.36	-0.02 (-0.07, 0.03)
Model 2	0.00	0.01 (-0.09, 0.12)	0.05 (-0.06, 0.17)	0.01 (-0.13, 0.14)	0.79	0.01 (-0.04, 0.06)
**FVC**						
Model 1	0.00	-0.03 (-0.14, 0.07)	-0.05 (-0.16, 0.06)	-0.10 (-0.23, 0.03)	0.12	-0.04 (-0.09, 0.01)
Model 5	0.00	-0.02 (-0.13, 0.09)	-0.02 (-0.14, 0.09)	-0.06 (-0.19, 0.08)	0.42	-0.02 (-0.07, 0.03)
**FEV_1/FVC ratio**						
Model 1	0.00	0.04 (-0.07, 0.15)	0.07 (-0.05, 0.18)	0.08 (-0.06, 0.21)	0.24	0.03 (-0.02, 0.08)
Model 2	0.00	0.05 (-0.06, 0.16)	0.11 (-0.01, 0.23)	0.13 (-0.01, 0.28)	0.04	0.06 (0.01, 0.12)
**$FEF_{25–75}$**						
Model 1	0.00	0.00 (-0.10, 0.11)	0.03 (-0.09, 0.14)	-0.01 (-0.14, 0.12)	0.97	-0.00 (-0.05, 0.05)
Model 2	0.00	0.01 (-0.10, 0.12)	0.07 (-0.05, 0.18)	0.05 (-0.09, 0.18)	0.40	0.03 (-0.03, 0.08)

* Linear trend was tested by treating the median values of quartiles as a continuous variable

FEV_1: forced expiratory volume in 1s; FVC: forced vital capacity; $FEF_{25–75}$: forced expiratory flow at 25–75% of FVC

Multivariable model 1: sex and total energy intake;

Multivariable model 2: further adjusted for maternal education, housing tenure at birth, financial difficulty during pregnancy, maternal ethnicity, index of multiple deprivation, rural living location, maternal and paternal history of atopic disease, older sibling, younger sibling, breastfeeding, maternal smoking, vigorous physical activity, history of food allergy, and season when the FFQ was completed.

Table S9: Linear regression coefficients (95% confidence interval) for post-bronchodilator lung function measures (z scores) at 15.5 years according to quartiles of dietary pattern score at 7 years, further adjusted for potential mediators

	Quartiles of dietary pattern score				P for trend*	Per SD
	Q1	Q2	Q3	Q4		
**'Health-conscious' dietary pattern**						
**FEV_1**						
+ TAC	0.00	0.08 (-0.06, 0.22)	0.09 (-0.05, 0.24)	0.17 (0.01, 0.33)	0.04	0.05 (-0.01, 0.10)
+ Vit C and E, carotene	0.00	0.08 (-0.06, 0.22)	0.09 (-0.05, 0.24)	0.18 (0.02, 0.33)	0.04	0.05 (-0.01, 0.11)
+ Zinc	0.00	0.08 (-0.06, 0.22)	0.09 (-0.06, 0.23)	0.16 (0.00, 0.31)	0.06	0.04 (-0.01, 0.10)
+ Selenium	0.00	0.08 (-0.06, 0.22)	0.09 (-0.06, 0.23)	0.15 (0.00, 0.31)	0.06	0.04 (-0.01, 0.10)
+ Vit D	0.00	0.08 (-0.06, 0.22)	0.09 (-0.06, 0.23)	0.16 (0.01, 0.32)	0.04	0.05 (-0.01, 0.10)
+ LC n-3 PUFA	0.00	0.07 (-0.07, 0.21)	0.08 (-0.07, 0.22)	0.15 (-0.01, 0.30)	0.07	0.04 (-0.02, 0.10)
+ Preformed vit A	0.00	0.07 (-0.07, 0.22)	0.08 (-0.06, 0.23)	0.16 (0.00, 0.31)	0.05	0.04 (-0.01, 0.10)
+ Fibre	0.00	0.07 (-0.07, 0.22)	0.09 (-0.06, 0.23)	0.16 (-0.00, 0.33)	0.06	0.05 (-0.01, 0.11)
+ BMI at 9y	0.00	0.06 (-0.08, 0.19)	0.08 (-0.06, 0.22)	0.15 (0.00, 0.30)	0.05	0.04 (-0.01, 0.10)
+ FMI at 9y	0.00	0.07 (-0.07, 0.21)	0.09 (-0.05, 0.23)	0.17 (0.01, 0.32)	0.03	0.05 (-0.01, 0.10)
**FVC**						
+ TAC	0.00	0.07 (-0.07, 0.21)	0.06 (-0.08, 0.20)	0.16 (0.01, 0.31)	0.05	0.05 (-0.00, 0.11)
+ Vit C and E, carotene	0.00	0.08 (-0.06, 0.22)	0.08 (-0.06, 0.22)	0.19 (0.04, 0.34)	0.02	0.07 (0.01, 0.12)
+ Zinc	0.00	0.07 (-0.06, 0.21)	0.06 (-0.08, 0.20)	0.16 (0.01, 0.31)	0.05	0.05 (0.00, 0.11)
+ Selenium	0.00	0.08 (-0.06, 0.21)	0.07 (-0.07, 0.21)	0.17 (0.03, 0.32)	0.03	0.06 (0.01, 0.11)
+ Vit D	0.00	0.08 (-0.06, 0.21)	0.07 (-0.06, 0.21)	0.18 (0.04, 0.33)	0.02	0.06 (0.01, 0.12)
+ LC n-3 PUFA	0.00	0.07 (-0.07, 0.20)	0.06 (-0.08, 0.20)	0.16 (0.01, 0.31)	0.04	0.06 (0.00, 0.11)
+ Preformed vit A	0.00	0.08 (-0.06, 0.21)	0.07 (-0.07, 0.21)	0.18 (0.03, 0.32)	0.02	0.06 (0.01, 0.11)
+ Fibre	0.00	0.06 (-0.07, 0.20)	0.06 (-0.09, 0.20)	0.15 (-0.00, 0.31)	0.07	0.05 (-0.00, 0.11)
+ BMI at 9y	0.00	0.06 (-0.08, 0.19)	0.07 (-0.07, 0.20)	0.17 (0.03, 0.31)	0.02	0.06 (0.01, 0.11)
+ FMI at 9y	0.00	0.07 (-0.06, 0.20)	0.08 (-0.06, 0.21)	0.19 (0.04, 0.33)	0.01	0.07 (0.02, 0.12)
**'Processed' dietary pattern**						
**FEV_1**						
+ TAC	0.00	0.04 (-0.09, 0.18)	-0.03 (-0.18, 0.11)	-0.12 (-0.29, 0.05)	0.12	-0.04 (-0.10, 0.03)
+ Vit C and E, carotene	0.00	0.02 (-0.11, 0.16)	-0.06 (-0.20, 0.09)	-0.16 (-0.33, 0.02)	0.06	-0.05 (-0.13, 0.02)
+ Zinc	0.00	0.05 (-0.09, 0.18)	-0.03 (-0.18, 0.11)	-0.11 (-0.29, 0.06)	0.16	-0.03 (-0.11, 0.04)
+ Selenium	0.00	0.05 (-0.08, 0.18)	-0.02 (-0.17, 0.12)	-0.10 (-0.27, 0.07)	0.21	-0.03 (-0.10, 0.05)
+ Vit D	0.00	0.04 (-0.09, 0.18)	-0.04 (-0.18, 0.10)	-0.12 (-0.29, 0.04)	0.11	-0.04 (-0.11, 0.03)
+ LC n-3 PUFA	0.00	0.05 (-0.08, 0.18)	-0.03 (-0.17, 0.11)	-0.11 (-0.27, 0.06)	0.17	-0.03 (-0.10, 0.03)
+ Preformed vit A	0.00	0.05 (-0.08, 0.19)	-0.02 (-0.17, 0.12)	-0.10 (-0.26, 0.07)	0.21	-0.03 (-0.10, 0.04)
+ Fibre	0.00	0.04 (-0.09, 0.18)	-0.04 (-0.18, 0.11)	-0.11 (-0.29, 0.06)	0.16	-0.03 (-0.11, 0.04)
+ BMI at 9y	0.00	0.05 (-0.08, 0.18)	-0.02 (-0.16, 0.12)	-0.12 (-0.28, 0.04)	0.13	-0.04 (-0.11, 0.03)
+ FMI at 9y	0.00	0.04 (-0.09, 0.18)	-0.04 (-0.18, 0.11)	-0.12 (-0.29, 0.04)	0.11	-0.04 (-0.11, 0.02)

**FVC**						
+ TAC	0.00	0.03 (-0.10, 0.15)	-0.05 (-0.19, 0.09)	-0.16 (-0.32, 0.00)	0.04	-0.04 (-0.11, 0.02)
+ Vit C and E, carotene	0.00	-0.00 (-0.13, 0.12)	-0.09 (-0.23, 0.06)	-0.21 (-0.38, -0.04)	0.01	-0.07 (-0.14, 0.00)
+ Zinc	0.00	0.04 (-0.09, 0.17)	-0.03 (-0.17, 0.11)	-0.11 (-0.28, 0.06)	0.17	-0.02 (-0.09, 0.05)
+ Selenium	0.00	0.03 (-0.10, 0.15)	-0.04 (-0.18, 0.09)	-0.14 (-0.30, 0.03)	0.09	-0.03 (-0.10, 0.03)
+ Vit D	0.00	0.02 (-0.11, 0.14)	-0.06 (-0.20, 0.07)	-0.17 (-0.33, -0.01)	0.03	-0.05 (-0.12, 0.01)
+ LC n-3 PUFA	0.00	0.02 (-0.10, 0.15)	-0.05 (-0.19, 0.09)	-0.15 (-0.31, 0.01)	0.05	-0.04 (-0.11, 0.02)
+ Preformed vit A	0.00	0.02 (-0.11, 0.15)	-0.06 (-0.20, 0.08)	-0.16 (-0.32, 0.00)	0.04	-0.05 (-0.11, 0.02)
+ Fibre	0.00	0.03 (-0.10, 0.16)	-0.04 (-0.18, 0.10)	-0.13 (-0.30, 0.04)	0.10	-0.03 (-0.10, 0.04)
+ BMI at 9y	0.00	0.02 (-0.11, 0.14)	-0.05 (-0.18, 0.08)	-0.17 (-0.32, -0.01)	0.03	-0.06 (-0.12, 0.01)
+ FMI at 9y	0.00	0.02 (-0.11, 0.14)	-0.06 (-0.20, 0.07)	-0.17 (-0.33, -0.01)	0.02	-0.06 (-0.12, 0.01)

* Linear trend was tested by treating the median values of quartiles as a continuous variable

FEV_1: forced expiratory volume in 1s; FVC: forced vital capacity; $FEF_{25–75}$: forced expiratory flow at 25–75% of FVC; TAC: total antioxidant capacity; Vit: vitamin; LC n-3 PUFA: long-chain omega-3 polyunsaturated fatty acids; BMI: body mass index; FMI: fat mass index

Benchmark multivariable model (model 2) included sex, total energy intake, maternal education, housing tenure at birth, financial difficulty during pregnancy, maternal ethnicity, index of multiple deprivation, rural living location, maternal and paternal history of atopic disease, older sibling, younger sibling, breastfeeding, maternal smoking, vigorous physical activity, history of food allergy, and season when the FFQ was completed. Results presented in this table were further adjusted for items mentioned for each model separately.

Table S10: Linear regression coefficients (95% confidence interval) for post-bronchodilator lung function measures (z scores) at 15.5 years according to quartiles of dietary pattern score at 7 years, stratified by asthma status at 7.5 years

	Quartiles of dietary pattern score				P for	P for
	**Q1**	**Q2**	**Q3**	**Q4**	**trend***	**interaction**
**'Health-conscious' dietary pattern**						
**FEV_1**						
Without asthma	0.00	0.05 (-0.11, 0.21)	0.08 (-0.09, 0.24)	0.13 (-0.04, 0.30)	0.14	
With asthma	0.00	0.25 (-0.17, 0.66)	0.07 (-0.35, 0.49)	0.32 (-0.13, 0.77)	0.26	0.68
**FVC**						
Without asthma	0.00	0.05 (-0.10, 0.20)	0.05 (-0.10, 0.21)	0.15 (-0.02, 0.31)	0.07	
With asthma	0.00	0.23 (-0.16, 0.63)	0.08 (-0.33, 0.49)	0.28 (-0.15, 0.71)	0.31	0.75
**FEV_1/FVC ratio**						
Without asthma	0.00	0.02 (-0.12, 0.15)	0.01 (-0.12, 0.14)	-0.04 (-0.19, 0.10)	0.48	
With asthma	0.00	-0.13 (-0.50, 0.25)	0.03 (-0.35, 0.41)	-0.09 (-0.49, 0.31)	0.85	0.97
**$FEF_{25–75}$**						
Without asthma	0.00	0.08 (-0.05, 0.22)	0.05 (-0.09, 0.19)	0.07 (-0.07, 0.22)	0.51	
With asthma	0.00	0.11 (-0.27, 0.48)	0.03 (-0.35, 0.41)	0.27 (-0.13, 0.68)	0.21	0.32
**'Traditional' dietary pattern**						
**FEV_1**						
Without asthma	0.00	0.03 (-0.12, 0.18)	0.10 (-0.06, 0.25)	-0.06 (-0.22, 0.10)	0.65	
With asthma	0.00	0.08 (-0.35, 0.50)	0.04 (-0.36, 0.45)	0.07 (-0.38, 0.51)	0.80	0.81
**FVC**						
Without asthma	0.00	0.01 (-0.14, 0.15)	0.07 (-0.07, 0.22)	-0.05 (-0.20, 0.10)	0.68	
With asthma	0.00	0.16 (-0.25, 0.56)	-0.02 (-0.41, 0.36)	0.10 (-0.33, 0.53)	0.86	0.85
**FEV_1/FVC ratio**						
Without asthma	0.00	-0.01 (-0.14, 0.11)	-0.01 (-0.13, 0.12)	-0.04 (-0.18, 0.09)	0.55	
With asthma	0.00	-0.12 (-0.50, 0.26)	-0.14 (-0.50, 0.22)	-0.13 (-0.53, 0.27)	0.49	0.52
**$FEF_{25–75}$**						
Without asthma	0.00	0.03 (-0.10, 0.16)	0.03 (-0.10, 0.16)	-0.08 (-0.21, 0.06)	0.31	
With asthma	0.00	-0.10 (-0.49, 0.28)	-0.08 (-0.45, 0.28)	-0.04 (-0.45, 0.36)	0.83	0.92
**'Processed' dietary pattern**						
**FEV_1**						
Without asthma	0.00	0.04 (-0.11, 0.18)	-0.01 (-0.17, 0.15)	-0.05 (-0.23, 0.14)	0.56	
With asthma	0.00	0.04 (-0.35, 0.44)	-0.14 (-0.57, 0.30)	-0.47 (-0.98, 0.03)	0.06	0.03
**FVC**						
Without asthma	0.00	0.02 (-0.12, 0.16)	-0.05 (-0.20, 0.11)	-0.10 (-0.28, 0.07)	0.20	
With asthma	0.00	-0.09 (-0.47, 0.30)	-0.15 (-0.57, 0.27)	-0.52 (-1.01, -0.04)	0.04	0.06
**FEV_1/FVC ratio**						
Without asthma	0.00	-0.02 (-0.14, 0.10)	0.06 (-0.08, 0.19)	0.13 (-0.03, 0.28)	0.09	
With asthma	0.00	0.19 (-0.17, 0.55)	0.05 (-0.34, 0.45)	0.02 (-0.44, 0.47)	0.97	0.20
**$FEF_{25–75}$**						
Without asthma	0.00	-0.01 (-0.13, 0.12)	0.00 (-0.13, 0.14)	0.01 (-0.15, 0.17)	0.87	
With asthma	0.00	0.08 (-0.28, 0.44)	-0.10 (-0.50, 0.29)	-0.29 (-0.75, 0.17)	0.18	0.03

† In with and without asthma groups, sample sizes were 367 and 2387 for FEV_1 and 380 and 2505 for both FVC and FEF_{25-75}, respectively.
* Linear trend was tested by treating the median values of quartiles as a continuous variable
FEV_1: forced expiratory volume in 1s; FVC: forced vital capacity; $FEF_{25–75}$: forced expiratory flow at 25–75% of FVC; *GPX4*: glutathione peroxidase 4
Multivariable model: sex, total energy intake, maternal education, housing tenure at birth, financial difficulty during pregnancy, maternal ethnicity, index of multiple deprivation, rural living location, maternal and paternal history of atopic disease, older sibling, younger sibling, breastfeeding, maternal smoking, vigorous physical activity, history of food allergy, and season when the FFQ was completed.

Table S11: Linear regression coefficients (95% confidence interval) for post-bronchodilator lung function measures (z scores) at 15.5 years according to quartiles of dietary pattern score at 7 years, stratified by maternal smoking in childhood†

	Quartiles of dietary pattern score				P for	P for
	**Q1**	**Q2**	**Q3**	**Q4**	**trend***	**interaction**
**'Health-conscious' dietary pattern**						
**FEV_1**						
None	0.00	0.07 (-0.09, 0.23)	0.05 (-0.11, 0.21)	0.14 (-0.03, 0.31)	0.13	
1-14/day	0.00	0.04 (-0.45, 0.53)	0.06 (-0.47, 0.59)	-0.30 (-0.80, 0.19)	0.19	0.23
15+/day	0.00	0.37 (-0.29, 1.03)	0.51 (-0.14, 1.16)	1.02 (0.18, 1.87)	0.02	0.03
**FVC**						
None	0.00	0.08 (-0.07, 0.24)	0.03 (-0.12, 0.19)	0.18 (0.02, 0.35)	0.04	
1-14/day	0.00	0.05 (-0.41, 0.51)	0.14 (-0.36, 0.64)	-0.24 (-0.71, 0.23)	0.26	0.37
15+/day	0.00	0.28 (-0.29, 0.85)	0.50 (-0.07, 1.06)	0.60 (-0.09, 1.30)	0.05	0.17
**FEV_1/FVC ratio**						
None	0.00	-0.02 (-0.15, 0.11)	-0.00 (-0.14, 0.13)	-0.10 (-0.24, 0.05)	0.17	
1-14/day	0.00	-0.19 (-0.62, 0.24)	-0.14 (-0.60, 0.32)	0.01 (-0.43, 0.44)	0.82	0.91
15+/day	0.00	-0.07 (-0.58, 0.45)	0.02 (-0.49, 0.52)	0.47 (-0.19, 1.12)	0.19	0.38
**$FEF_{25–75}$**						
None	0.00	0.08 (-0.05, 0.21)	0.04 (-0.10, 0.17)	0.05 (-0.09, 0.20)	0.71	
1-14/day	0.00	-0.02 (-0.46, 0.41)	-0.05 (-0.53, 0.42)	-0.10 (-0.55, 0.34)	0.63	0.86
15+/day	0.00	0.28 (-0.29, 0.86)	0.26 (-0.31, 0.83)	0.89 (0.19, 1.59)	0.02	0.04
**'Traditional' dietary pattern**						
**FEV_1**						
None	0.00	0.04 (-0.11, 0.19)	0.08 (-0.07, 0.23)	-0.02 (-0.18, 0.13)	0.89	
1-14/day	0.00	0.32 (-0.14, 0.79)	0.51 (0.02, 0.99)	0.06 (-0.46, 0.57)	0.55	0.59
15+/day	0.00	-0.01 (-0.75, 0.72)	0.37 (-0.39, 1.13)	0.30 (-0.35, 0.96)	0.29	0.39
**FVC**						
None	0.00	0.06 (-0.08, 0.21)	0.08 (-0.06, 0.23)	0.02 (-0.13, 0.18)	0.71	
1-14/day	0.00	0.11 (-0.34, 0.55)	0.30 (-0.16, 0.76)	-0.13 (-0.62, 0.35)	0.84	0.67
15+/day	0.00	-0.11 (-0.73, 0.52)	0.15 (-0.48, 0.78)	0.18 (-0.38, 0.73)	0.46	0.69
**FEV_1/FVC ratio**						
None	0.00	-0.08 (-0.21, 0.05)	-0.07 (-0.20, 0.06)	-0.11 (-0.25, 0.02)	0.11	
1-14/day	0.00	0.18 (-0.23, 0.59)	0.18 (-0.25, 0.60)	0.11 (-0.34, 0.56)	0.58	0.14
15+/day	0.00	0.27 (-0.29, 0.84)	0.17 (-0.41, 0.76)	0.32 (-0.18, 0.82)	0.24	0.20
**$FEF_{25–75}$**						
None	0.00	-0.03 (-0.16, 0.10)	-0.06 (-0.18, 0.07)	-0.13 (-0.26, 0.01)	0.06	
1-14/day	0.00	0.28 (-0.14, 0.70)	0.39 (-0.05, 0.83)	0.18 (-0.28, 0.63)	0.33	0.09
15+/day	0.00	0.48 (-0.15, 1.10)	0.71 (0.08, 1.34)	0.32 (-0.24, 0.88)	0.23	0.04
**'Processed' dietary pattern**						
**FEV_1**						
None	0.00	0.06 (-0.09, 0.20)	-0.04 (-0.20, 0.12)	-0.08 (-0.26, 0.10)	0.28	
1-14/day	0.00	-0.18 (-0.68, 0.33)	0.11 (-0.41, 0.64)	-0.09 (-0.71, 0.54)	0.99	0.97
15+/day	0.00	0.44 (-0.30, 1.18)	-0.25 (-0.95, 0.46)	-0.48 (-1.34, 0.38)	0.16	0.33

**FVC**						
None	0.00	0.03 (-0.11, 0.17)	-0.06 (-0.21, 0.09)	-0.10 (-0.28, 0.08)	0.20	
1-14/day	0.00	-0.18 (-0.65, 0.29)	-0.06 (-0.55, 0.42)	-0.33 (-0.93, 0.26)	0.36	0.57
15+/day	0.00	0.18 (-0.45, 0.82)	-0.34 (-0.94, 0.27)	-0.59 (-1.31, 0.13)	0.05	0.15
**FEV_1/FVC ratio**						
None	0.00	0.01 (-0.11, 0.14)	0.05 (-0.08, 0.18)	0.07 (-0.08, 0.22)	0.33	
1-14/day	0.00	-0.08 (-0.52, 0.35)	0.29 (-0.17, 0.74)	0.39 (-0.15, 0.93)	0.08	0.33
15+/day	0.00	0.42 (-0.16, 0.99)	0.34 (-0.20, 0.89)	0.53 (-0.13, 1.20)	0.15	0.24
**$FEF_{25–75}$**						
None	0.00	0.03 (-0.09, 0.15)	-0.01 (-0.14, 0.13)	-0.02 (-0.17, 0.14)	0.76	
1-14/day	0.00	-0.11 (-0.55, 0.33)	0.13 (-0.33, 0.59)	0.01 (-0.55, 0.57)	0.77	0.87
15+/day	0.00	0.29 (-0.36, 0.95)	-0.19 (-0.81, 0.43)	-0.23 (-0.97, 0.51)	0.35	0.97

† In none, 1-14/day, and 15+/day groups, sample sizes were 2,393, 274, and 161 for FEV_1 and 2,498, 289, and 174 for both FVC and FEF_{25-75}, respectively.

* Linear trend was tested by treating the median values of quartiles as a continuous variable

FEV_1: forced expiratory volume in 1s; FVC: forced vital capacity; $FEF_{25–75}$: forced expiratory flow at 25–75% of FVC

Multivariable model: sex, total energy intake, maternal education, housing tenure at birth, financial difficulty during pregnancy, maternal ethnicity, index of multiple deprivation, rural living location, maternal and paternal history of atopic disease, older sibling, younger sibling, breastfeeding, maternal smoking, vigorous physical activity, history of food allergy, and season when the FFQ was completed.

Table S12: Linear regression coefficients (95% confidence interval) for post-bronchodilator lung function measures (z scores) at 15.5 years according to quartiles of the health-conscious dietary pattern score at 7 years, stratified by *GPX4*† genotype (rs713041)

	Quartiles of dietary pattern score				P for	P for
	Q1	Q2	Q3	Q4	trend*	interaction
**'Health-conscious' dietary pattern**						
**FEV_1**						
Genotype CC	0.00	0.12 (-0.16, 0.40)	-0.03 (-0.32, 0.25)	-0.04 (-0.34, 0.26)	0.54	
Genotype CT	0.00	0.08 (-0.15, 0.31)	0.03 (-0.20, 0.26)	0.22 (-0.02, 0.46)	0.08	0.01
Genotype TT	0.00	0.10 (-0.30, 0.50)	0.39 (-0.01, 0.78)	0.30 (-0.15, 0.74)	0.14	0.06
**FVC**						
Genotype CC	0.00	0.12 (-0.15, 0.38)	0.02 (-0.26, 0.29)	0.05 (-0.24, 0.33)	0.97	
Genotype CT	0.00	0.13 (-0.09, 0.35)	0.03 (-0.19, 0.25)	0.26 (0.02, 0.49)	0.05	0.04
Genotype TT	0.00	0.02 (-0.35, 0.40)	0.23 (-0.14, 0.61)	0.23 (-0.19, 0.65)	0.20	0.09
**FEV_1/FVC ratio**						
Genotype CC	0.00	0.05 (-0.18, 0.28)	-0.05 (-0.29, 0.19)	-0.15 (-0.40, 0.11)	0.15	
Genotype CT	0.00	-0.09 (-0.29, 0.11)	0.03 (-0.17, 0.23)	-0.09 (-0.30, 0.13)	0.60	0.46
Genotype TT	0.00	0.04 (-0.28, 0.37)	0.08 (-0.24, 0.40)	0.09 (-0.27, 0.45)	0.62	0.43
**$FEF_{25–75}$**						
Genotype CC	0.00	0.05 (-0.18, 0.28)	-0.09 (-0.33, 0.15)	-0.21 (-0.46, 0.04)	0.04	
Genotype CT	0.00	0.09 (-0.11, 0.29)	0.07 (-0.13, 0.27)	0.19 (-0.02, 0.40)	0.10	0.001
Genotype TT	0.00	0.27 (-0.07, 0.61)	0.22 (-0.11, 0.56)	0.26 (-0.12, 0.65)	0.29	0.04

† In CC, CT, and TT groups, sample sizes were 747, 1131, and 467 for FEV_1 and 778, 1186, and 493 for FVC and FEF_{25-75}, respectively.

* Linear trend was tested by treating the median values of quartiles as a continuous variable

FEV_1: forced expiratory volume in 1s; FVC: forced vital capacity; $FEF_{25–75}$: forced expiratory flow at 25–75% of FVC; *GPX4*: glutathione peroxidase 4

Multivariable model: sex and total energy intake; further adjusted for maternal education, housing tenure at birth, financial difficulty during pregnancy, maternal ethnicity, index of multiple deprivation, rural living location, maternal and paternal history of atopic disease, older sibling, younger sibling, breastfeeding, maternal smoking, vigorous physical activity, history of food allergy, and season when the FFQ was completed.

Table S13: Odds ratio (95% confidence interval) for incident asthma at 11 or 14 years according to quartiles of dietary pattern score at 7 years, adjusted for additional factors as well as exclusions (sensitivity analysis)

	Quartiles of dietary pattern score				P for	Per SD
	Q1	Q2	Q3	Q4	trend*	
**'Health-conscious' dietary pattern**						
Cases/non-cases	86/984	94/1043	102/1081	109/1051		
Model 3	1.00	0.99 (0.72-1.35)	1.02 (0.74-1.40)	1.04 (0.74-1.47)	0.78	1.01 (0.89, 1.14)
Model 4	1.00	1.02 (0.75-1.41)	1.08 (0.78-1.50)	1.11 (0.77-1.60)	0.53	1.03 (0.91, 1.18)
Model 5	1.00	1.02 (0.70-1.50)	1.07 (0.73-1.56)	0.97 (0.65-1.46)	0.87	1.00 (0.87, 1.16)
Model 6	1.00	1.00 (0.73-1.37)	1.03 (0.75-1.42)	1.04 (0.74-1.47)	0.77	1.00 (0.89, 1.13)
Model 7	1.00	1.01 (0.73-1.39)	1.09 (0.79-1.50)	1.10 (0.78-1.55)	0.54	1.03 (0.91, 1.16)
Model 8	1.00	1.01 (0.73-1.41)	1.07 (0.77-1.50)	1.12 (0.79-1.60)	0.47	1.03 (0.91, 1.17)
**'Traditional' dietary pattern**						
Cases/non-cases	90/1085	101/1040	103/1009	97/1025		
Model 3	1.00	1.20 (0.88-1.63)	1.23 (0.90-1.68)	1.09 (0.78-1.53)	0.60	1.01 (0.90, 1.14)
Model 4	1.00	1.19 (0.88-1.62)	1.24 (0.91-1.69)	1.07 (0.77-1.49)	0.62	1.02 (0.90, 1.14)
Model 5	1.00	1.26 (0.88-1.82)	1.17 (0.80-1.69)	1.21 (0.82-1.78)	0.41	1.04 (0.90, 1.19)
Model 6	1.00	1.20 (0.89-1.63)	1.24 (0.91-1.68)	1.11 (0.80-1.53)	0.51	1.02 (0.91, 1.14)
Model 7	1.00	1.19 (0.88-1.62)	1.22 (0.90-1.66)	1.10 (0.79-1.52)	0.54	1.02 (0.91, 1.15)
Model 8	1.00	1.23 (0.90-1.69)	1.19 (0.86-1.64)	1.15 (0.82-1.61)	0.47	1.02 (0.90, 1.16)
**'Processed' dietary pattern**						
Cases/non-cases	107/1126	104/1073	92/1028	88/932		
Model 3	1.00	1.00 (0.74-1.35)	0.96 (0.69-1.33)	0.91 (0.62-1.34)	0.61	1.04 (0.89, 1.22)
Model 4	1.00	1.00 (0.74-1.34)	0.95 (0.69-1.31)	0.89 (0.62-1.30)	0.53	1.02 (0.88, 1.18)
Model 5	1.00	0.95 (0.67-1.35)	0.83 (0.57-1.22)	0.77 (0.50-1.20)	0.21	0.98 (0.82, 1.16)
Model 6	1.00	0.99 (0.74-1.33)	0.94 (0.68-1.29)	0.88 (0.61-1.26)	0.45	1.01 (0.87, 1.17)
Model 7	1.00	1.00 (0.74-1.34)	0.95 (0.69-1.31)	0.88 (0.60-1.27)	0.47	1.01 (0.87, 1.18)
Model 8	1.00	0.94 (0.69-1.28)	0.91 (0.66-1.27)	0.82 (0.56-1.20)	0.31	0.98 (0.83, 1.14)

* Linear trend was tested by treating the median values of quartiles as a continuous variable

Benchmark model (multivariable model 2): sex, total energy intake, maternal education, housing tenure at birth, financial difficulty during pregnancy, maternal ethnicity, index of multiple deprivation, rural living location, maternal and paternal history of atopic disease, older sibling, younger sibling, breastfeeding, maternal smoking, vigorous physical activity, history of food allergy, and season when the FFQ was completed;

Multivariable model 3: further adjusted for the other two dietary patterns (mutual adjustment);

Multivariable model 4: further adjusted for the corresponding maternal dietary pattern in pregnancy;

Multivariable model 5: further adjusted for atopy (by skin prick test; n=3,311);

Multivariable model 6: further adjusted for any supplement use;

Multivariable model 7: model 2 excluding children of non-white mothers;

Multivariable model 8: model 2 excluding children with extreme energy intakes.
